# Genome-Wide Investigation of Apyrase (APY) Genes in Peanut (*Arachis hypogaea* L.) and Functional Characterization of a Pod-Abundant Expression Promoter *AhAPY2-1p*

**DOI:** 10.3390/ijms24054622

**Published:** 2023-02-27

**Authors:** Yasir Sharif, Gandeka Mamadou, Qiang Yang, Tiecheng Cai, Yuhui Zhuang, Kun Chen, Ye Deng, Shahid Ali Khan, Niaz Ali, Chong Zhang, Ali Raza, Hua Chen, Rajeev K. Varshney, Weijian Zhuang

**Affiliations:** 1 College of Agriculture, Center of Legume Plant Genetics and System Biology, Institute of Oil Crops, Fujian Agriculture and Forestry University (FAFU), Fuzhou 350002, China; 2 College of Life Science, Fujian Agriculture and Forestry University, Fuzhou 350002, China; 3 College of Plant Protection, Fujian Agriculture and Forestry University, Fuzhou 350002, China; 4 State Agricultural Biotechnology Centre, Crop & Food Innovation Centre, Food Futures Institute, Murdoch University, Murdoch, WA 6150, Australia

**Keywords:** *cis*-elements, environmental stress, functional annotation, GUS activity, miRNAs, pericarp specific, phylogenetic analysis

## Abstract

Peanut (*Arachis hypogaea* L.) is an important food and feed crop worldwide and is affected by various biotic and abiotic stresses. The cellular ATP levels decrease significantly during stress as ATP molecules move to extracellular spaces, resulting in increased ROS production and cell apoptosis. Apyrases (*APYs*) are the nucleoside phosphatase (NPTs) superfamily members and play an important role in regulating cellular ATP levels under stress. We identified 17 *APY* homologs in *A. hypogaea* (*AhAPYs*), and their phylogenetic relationships, conserved motifs, putative miRNAs targeting different *AhAPYs*, *cis*-regulatory elements, etc., were studied in detail. The transcriptome expression data were used to observe the expression patterns in different tissues and under stress conditions. We found that the *AhAPY2-1* gene showed abundant expression in the pericarp. As the pericarp is a key defense organ against environmental stress and promoters are the key elements regulating gene expression, we functionally characterized the *AhAPY2-1* promoter for its possible use in future breeding programs. The functional characterization of *AhAPY2-1P* in transgenic *Arabidopsis* plants showed that it effectively regulated *GUS* gene expression in the pericarp. GUS expression was also detected in flowers of transgenic *Arabidopsis* plants. Overall, these results strongly suggest that *APYs* are an important future research subject for peanut and other crops, and *AhPAY2-1P* can be used to drive the resistance-related genes in a pericarp-specific manner to enhance the defensive abilities of the pericarp.

## 1. Introduction

Peanut, or groundnut (*Arachis hypogaea* L.), is an important food legume of tropical and subtropical countries. It is the primary source of edible oil and proteins and the staple food of many African and Asian countries [1]. Peanut is cultivated in more than 100 countries across the world, while China, India, Nigeria, the United States, and Sudan are leading peanut-producing countries [2]. Botanically, peanut is a unique plant among legumes due to the pegging phenomenon [3]. It produces flowers above ground, and after pollination, the gynophore enters the soil and produce seeds underground [3,4]. As it bore seeds beneath the soil, seeds are always prone to attack by soil-borne fungal and bacterial pathogens. These pathogens mainly deteriorate peanut yield and quality [5]. The introduction of stress-resistant peanut cultivars could potentially solve the issue [6]. Investigating the stress-resistant mechanism and characterizing stress-responsive genes is key for any successful transgenic breeding program. In recent years, the genomes of many crop species have been worked out [7,8,9], and with the help of bioinformatics tools, genome-wide systematic studies of stress-related gene families are available. Similarly, the genome information of cultivated peanut [10,11] and its diploid progenitors [12] is available now. In peanut, the genome-wide studies for some gene/transcription factor families, including WRKY [13], bHLH [14], bZIP [15], GRF [16], ARF [17], APX [18], AP2/ERF [19], etc., have been performed, and their stress-responsive roles have been elucidated. Identifying more stress-responsive gene/protein families and incorporating them into resistance mechanisms could improve stress resistance in peanut.

Extracellular ATP levels significantly increase in response to environmental stress, which initiates cell death and apoptosis [20]. The GDA1-CD39 nucleoside phosphatase/Apyrases family is ubiquitously found in animals, plants, bacteria, and fungi, and regulates cellular ATP levels [21,22,23,24]. APYRASEs (*APYs*) are a class of nucleoside triphosphate (NTP) diphosphohydolases (NTPDases) that maintain cellular NTP homeostasis by removing terminal phosphatases from NTPs and DTPs [21,25]. Based on subcellular localization, Apyrases are divided into two categories ecto- and endo-apyrases [26]. Endo-apyrases are usually localized in intracellular vesicles, endoplasmic reticulum (ER), and Golgi apparatus, while ecto-apyrases are present on the cell surface [27]. APYs, in contrast to ATPases, can use a variety of cofactors, including calcium Ca^2+^, magnesium Mg^2+^, manganese Mn^2+^, and zinc Zn^2+^, while ATPases use only Mg^2+^ as a cofactor [28]. The cellular ATP is not only the source of energy, but it also mediates different cellular mechanisms during stress, including potassium K^+^ homeostasis, vacuole Na^+^ distribution, Na^+^/H^+^ exchange, K^+^/Na^+^ exchange under salt stress [29], regulation of reactive oxygen species (ROS), and plasma membrane repairs [30]. Thus APYs/NTPs are essential in maintaining cellular ATP homeostasis for a plant’s normal functioning under stressed conditions.

Human *APYs* are well-characterized, while among plants, *APYs* are well-described in *Arabidopsis*. Seven *APYs* have been identified in *Arabidopsis*, which are further divided into three classes based on their functions [25]. *Arabidopsis APY1* and *APY2* are located within the Golgi apparatus and play roles in root development, stomata opening/closure, and pollen development [31,32]. *AtAPY1* and *AtAPY2* are endo-*APYs*, but their mutations can increase extracellular ATP levels [33]. The biochemical and physiological functions of *AtAPY3*-*AtAPY5* have not been worked out. Some initial studies for *AtAPY6* and *AtAPY7* are available; *AtAPY6* and *AtAPY7* are also involved in pollen development [34]. It is evident from recent studies that the *APYs* play a role in plant defense mechanisms. *APYs* are involved in plant defense against different pathogenic organisms, including fungal pathogens resistance [35], drought stress tolerance [36], salt stress tolerance [37], and waterlogging tolerance [38]. However, the molecular mechanisms responsible for the defense responses are still unclear.

The advancements in modern biotechnological techniques have made bioinformatics work easy. Modern genotyping methods have revealed the genetic atlas of many important crop/plant species. A piece of comprehensive information on many quality traits, population structure, genetic diversity, etc., is available for peanut [39,40,41]. It is evident from previous studies that *APYs* play key roles against biotic and abiotic stress factors in plants. Functional studies of *APYs* in crop plants could provide new insights for stress breeding. Based on the studies mentioned above, we hypothesized that *APYs* in the peanut genome could be investigated for their potential involvement in stress-related or quality traits. So, we performed a comprehensive analysis to identify the *APYs* in the peanut genome. Fast and accurate sequencing methods have resulted in the availability of many transcriptome datasets for yield and stress-responsive traits [42].

Similarly, the QTL and fine-mapping techniques have generated a handsome amount of data for various quality- and stress-related traits [43,44,45]. The transcriptome profiles of cultivated peanut suggested that the *AhAPY2-1* gene could be a potential candidate for pericarp-specific expression. The pericarp is the first organ that protects edible seeds from biotic and abiotic stresses. Several studies are available for pod/pericarp development, but there is a lack of work on the resistance-related behavior of the pericarp. We selected the pericarp-specific promoter for further study. Promoters are key regions to guide and regulate a gene’s expression. We cloned the promoter region of the *AhAPY2-1* gene and functionally characterized it in transgenic *Arabidopsis* plants. This study first reported the Apyrases/Nucleotide phosphatases proteins in the genome of cultivated peanut and characterized a pericarp abundant expression promoter in peanut. We are convinced that this study will enhance the understanding of peanut apyrases and provide a base for further research.

## 2. Results

### 2.1. Identification, Genomic Locations, and Physicochemical Properties of AhAPYs

**Figure 1:**
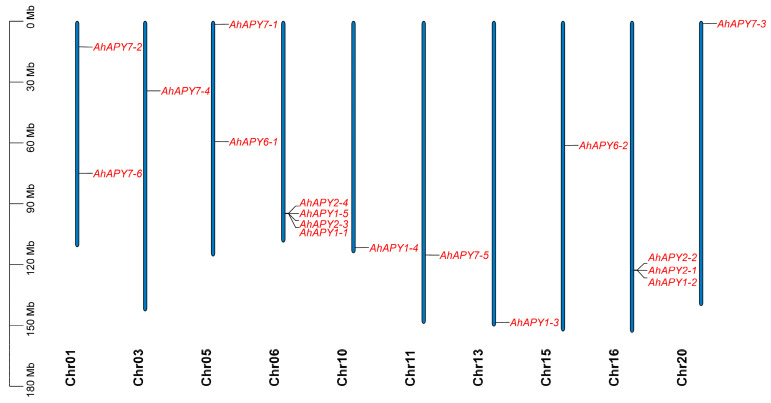
Chromosomal positions of *AhAPYs*. Most of the chromosomes possessed a single *APY*, while Chr06 and Chr16 had four and three *APYs*, respectively. Chr02, Chr04, Chr7-Chr9, Chr12, Chr14, and Chr17-Chr19 did not possess any *APY*.

The APYs/NTPs genes in the genome of cultivated peanut were identified stepwise. Protein sequences of seven *Arabidopsis* apyrases were used as queries to search the apyrases in diploid peanut species. The BLAST results revealed the presence of eight *APYs* homologs in the genomes of *A. duranensis* and *A. ipaensis* each (Table S2), and 17 genes were found in the cultivated peanut genome (Table 1). The apyrases of *A. duranensis*, *A. ipaensis*, and *A. hypogaea* were named based on the phylogenetic relationships with *Arabidopsis* apyrases. The chromosomal distribution analysis of *AhAPYs* showed their uneven distribution in the genome. Chromosomes 2, 4, 7–9, 12, 14, 17–19 did not possess any apyrases. Chromosome 6 possessed four *APYs*, and chromosome 16 possessed three *APYS*. Chromosomes 1 and 5 possessed two *APYs* each, while all remaining chromosomes (3, 10, 11, 13, 15, and 20) possessed one *APYs* each (Figure 1). The chromosomal distribution of *A. duranensis* and *A. ipaensis* are presented in Figure S1. All *AhAPYs* possessed varying physicochemical properties. The subcellular localization prediction showed that most proteins are located in the plasma membrane, with few in the cytoplasm, chloroplast, mitochondria, extracellular spaces, nucleus, and vacuole. The theoretical isoelectric points of AhAPYs varied from 4.69 (*AhAPY2-4*) to 9.94 (*AhAPY7-4*), and molecular weight ranged from 14.96 KDa (*AhAPY2-4*) to 82.39 KDa (*AhAPY7-1*) (Table 1). The physicochemical properties, protein, CDS lengths, and the number of exons in *AdAPYs* and *AiAPYs* are given in Table S2.

### 2.2. Gene Structure, Conserved Motifs, and Protein 3D Structure Simulation

**Figure 2:**
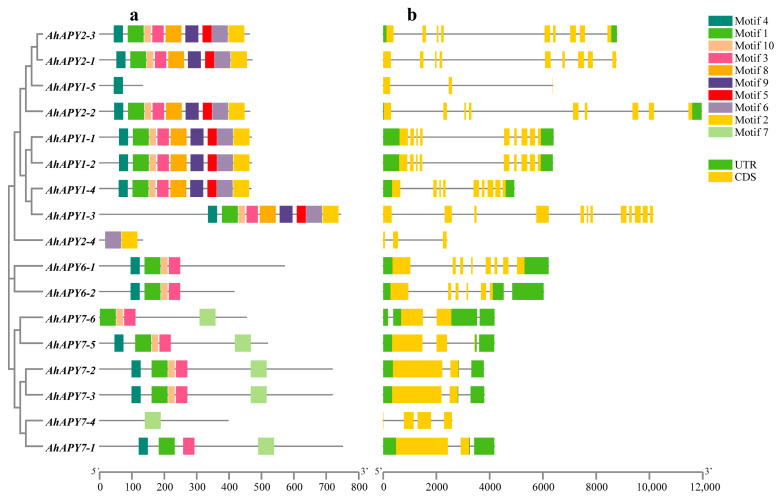
Conserved motifs and gene structure analysis of *AhAPYs*: (**a**) top 10 conserved motifs of AhAPYs predicted by MEME suite; (**b**) exon-intron distribution patterns of *AhAPYs*. The legends are given on the right side.

Structurally, all *AhAPYs* were diverse, and they possessed a different number of exons. Exon numbers varied from 2 to 12 exons, while 9 exons were common among *AhAPYs* (Figure 2). *AhAPYs* possessed varying genomic, CDS, and protein lengths, as *AhAPY2-2* is the largest gene, comprising 11,962 bp long genomic sequence, 1395 bp long CDS, and 464 aa protein length, while the *AhAPY2-4* is the smallest gene with genomic sequence of 2387 bp. Interestingly, *AhAPY7-1* possessed the longest CDS and protein length of 2253 bp and 750 aa, respectively (Table 1). Conserved motif analysis revealed the presence of common and unique motifs in *AhAPY* genes. Proteins sharing common motifs tend to crowd in the same group, indicating their similar functions. The 1st, 3rd, 4th, and 10th motifs were found in most *AhAPYs*. *AhAPY1-5* and *AhAPY7-4* possessed only one motif each (Figure 2). Gene structures and conserved motif patterns of *A. duranensis APYs* are given in Figure S2, and of *A. ipaensis* are shown in Figure S3. Simulations of 3D modules of tertiary protein structures of 17 *AhAPYs* showed that an extended strand linked the similar subunits that are further surrounded by an alpha helix. Overall, this arrangement represents a characteristic feature of the GDA1-CD39 NPT superfamily (Figure S4). Previous studies on *Arabidopsis APYs* showed that *AtAPY1* and *AtAPY2* perform similar functions, and *AtAPY6* and *AtAPY7* perform similar functions; the 3D structures of *AhAPYs* represent the structural similarity of the first group with the second, and the sixth group with the seventh. This fact is also evident from the conserved motif analysis, which divides the peanut *APYs* into three groups.

### 2.3. Phylogenetic and Gene Duplication Analysis

**Figure 3:**
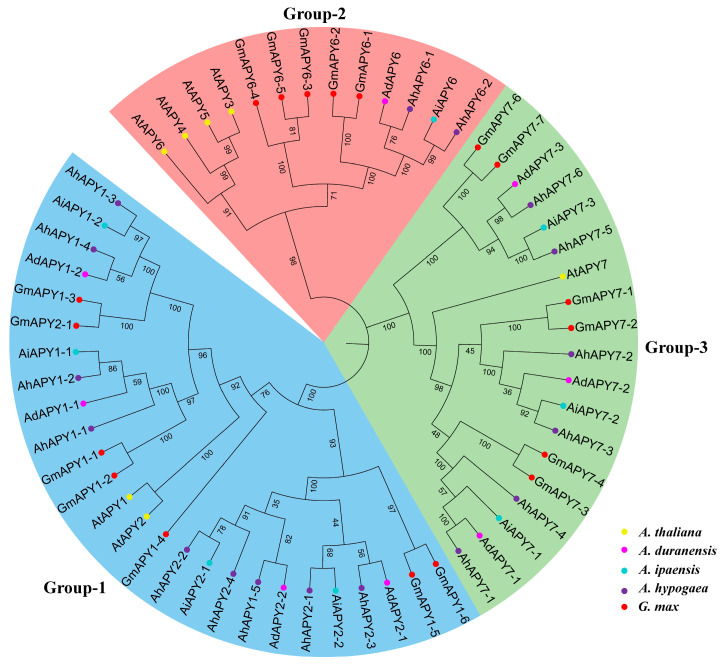
The phylogenetic relationships of all *AhAPYs* with their diploid parents and model legumes. The phylogenetic tree was constructed with the APYs homologs in *A. thaliana*, *A. hypogaea*, *A. duranensis*, *A. ipaensis*, and *G. max*. The phylogenetic tree divided all *APYs* into three groups. Numbers at the nodes represents percentage bootstrap values and different background colors represents different phylogenetic groups.

**Figure 4:**
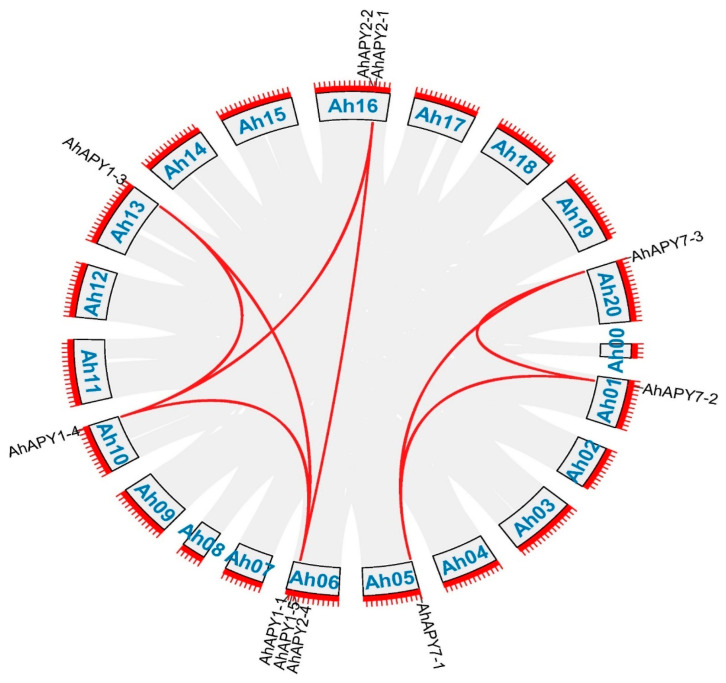
Duplicated gene pairs among *AhAPYs*. Out of 17 *AhAPYs*, 14 *AhAPYs* are in the form of duplicated genes, making seven duplicated pairs. All duplicated genes are segmentally duplicated. The red lines show the duplicated gene pairs, while the gray lines in the background show the syntenic blocks (duplicated pairs) among different chromosomes.

To study the evolutionary relationships of cultivated peanut *APYs* with their wild relatives and model legume crops, a phylogenetic tree was constructed among the protein sequences of *A. hypogaea* and their homologs in *A. duranensis*, *A. ipaensis*, *A. thaliana*, and *G. max*. The phylogenetic tree divided all the *APYs* into three main groups. *APY1* and *APY2* of all species tended to cluster in the same group. *APY6* of all species clustered in a separate group, and members of *APY7* from all species clustered in a separate group (Figure 3). Phylogenetic grouping provided evidence of solid evolutionary relationships among these species. Gene duplication is a major force behind genome evolution. Duplicated gene pairs among *AhAPYs* were identified by their phylogenetic relationships. Among 17 *AhAPYs*, seven duplicated gene pairs were found (Figure 4). The Ks and Ka values for each duplicated pair were calculated by a simple Ka/Ks calculator available at TBtools. Evolutionary rates (Ka/Ks ratio) for each duplicated gene pair were calculated. The Ka/Ks = 1 was considered neutral selection pressure, Ka/Ks > 1 was regarded as positive selection pressure, and Ka/Ks < 1 was considered purifying selection pressure [46]. Ka/Ks values of all gene pairs showed that purifying selection pressure was mainly involved in the duplication process (Table 2). The expected divergence time for duplicated pairs varied from 1.26 Mya (million years ago) for the gene pair *AhAPY7-2:AhAPY7-3* to 127.405 Mya for *AhAPY1-5:AhAPY1-4* (Table 2).

### 2.4. Prediction of miRNAs Targeting AhAPYs and Analysis of Putative Protein–Protein Interactions

**Figure 5:**
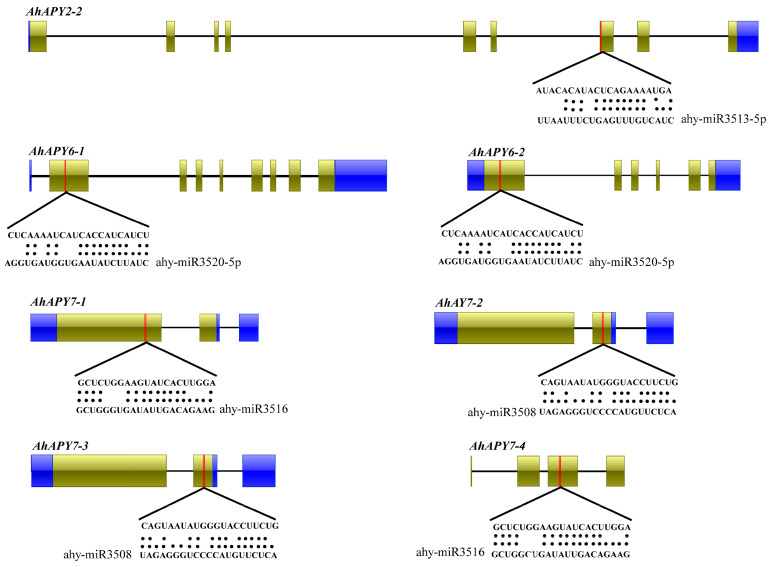
Putative miRNAs targeting the *AhAPYs*. The red color in exons shows the miRNA target sites. miRNAs from four different families targeted seven *AhAPYs* out of 17.

Non-coding miRNAs, as the key regulators of post-transcriptional gene regulation, have attracted the attention of many researchers. Some studies have reported their role in biotic and abiotic stress responses [47,48]. To illustrate the possible miRNAs involved in regulating the peanut APYs, we predicted the miRNAs targeting the AhAPYs through the online miRNAs database, the PsRNATarget database. We found four different miRNAs targeting seven peanut APYs (Table S3). The ahy-miR3508 targeted *AhAPY7-2* and *AhAPY7-3*; ahy-miR3513-5p targeted *AhAPY2-2*; ahy-miR3516 targeted *AhAPY7-1* and *AhAPY7-4*; ahy-miR3520-5p targeted two genes, *AhAPY6-1* and *AhAPY6-2*. These predicted miRNAs and their target sites in the CDS region are shown in Figure 5. These miRNAs provide future research dimensions for functional validation of their expression levels and role in gene regulation.

To understand the putative functions of AhAPYs, the protein interaction network analysis was performed based on APYs orthologs in *Arabidopsis* using the STRING database. The top 10 interactions were considered with a high threshold level (0.7). The interaction network prediction showed that *AhAPY1-5* has functions related to *Arabidopsis* ZEU1 protein, *Arabidopsis* ADSS (Adenylosuccinate synthetase, chloroplastic), Fac1 (AMP deaminase), and some other proteins (Figure S5). Protein *AhAPY6-2* showed strong interactions with *Arabidopsis* ADSS, ZEU1, FAC1, THY1 (Bifunctional dihydrofolate reductase-thymidylate synthase 1), THY2 (Bifunctional dihydrofolate reductase-thymidylate synthase 2), and with other *Arabidopsis* proteins. *AhAPY7-6* showed interaction with PUM10 (Putative pumilio homolog 10). Multiple sequence search methods showed that other AhAPYs did not interact with *Arabidopsis* proteins. Their interactions need more work, or there is a possibility that these proteins have some special functions that are not exploited well.

### 2.5. Analysis of Cis-Regulatory Elements

**Figure 6:**
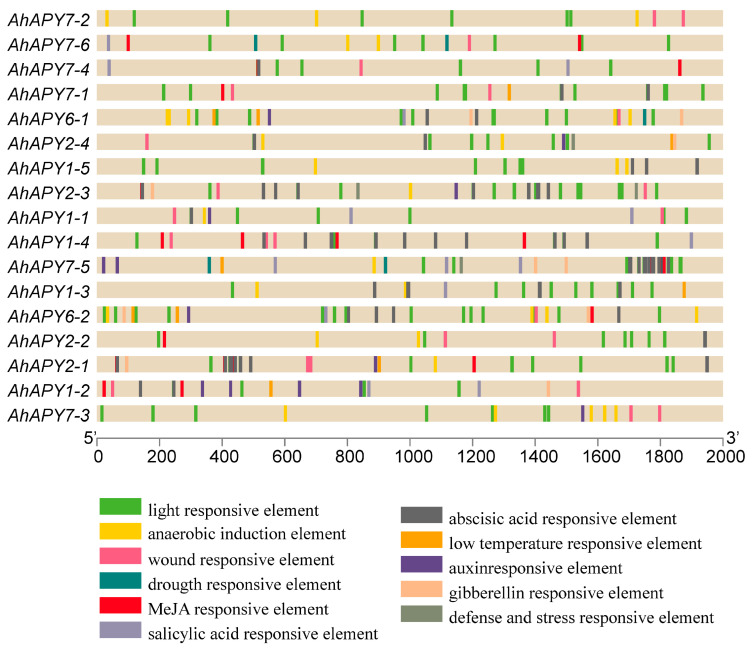
*Cis*-regulatory elements in the promoter regions (2 kb upstream of the start codon) of *AhAPYs*. *Cis*-elements analysis revealed that promoter regions of *AhAPYs* possessed important elements responsive to light, phytohormones, defense and stress, low temperature, and wound responsiveness.

Analyzing transcription factor binding sites or *cis*-regulatory elements is key for functional genomic studies. We searched the 2 kb upstream regions of promoters to find out the *cis*-regulatory elements to predict the possible functions of *AhAPYs*. Aside from the core promoter elements (TATA box, CAAT box), other important regulatory elements were also found in the promoter regions. Other important *cis*-elements mainly included light-responsive elements (G-box, Box 4, GATA-motif), hormone-responsive elements (abscisic acid, ABRE; salicylic acid, SARE; methyl jasmonate, MeJA; gibberellin, GBRE), growth- and development-related (anaerobic induction, ARE; zein metabolism, O2-site; meristem expression; CAT-box), and stress-responsive elements (wound responsive elements, WUN motif; low temperature responsive, LTR; defense responsive; TC-rich repeats). The detail of *cis*-regulatory elements and their positions in the promoter regions are shown in Figure 6.

### 2.6. Synteny and Functional Annotation Analysis

**Figure 7:**
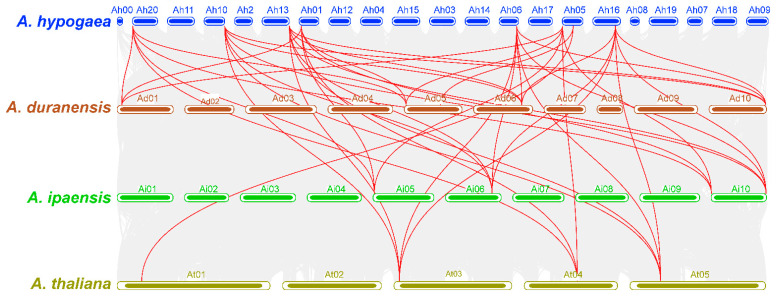
Synteny analysis of *A. hypogaea*, *A. duranensis*, *A. ipaensis*, and *A. thaliana*. Grey lines show the collinear regions of *A. hypogaea* with other species. Red lines represent the *AhAPYs*. *AhAPYs* on both subgenomes established syntenic relations with its diploid parents and *Arabidopsis*.

**Figure 8:**
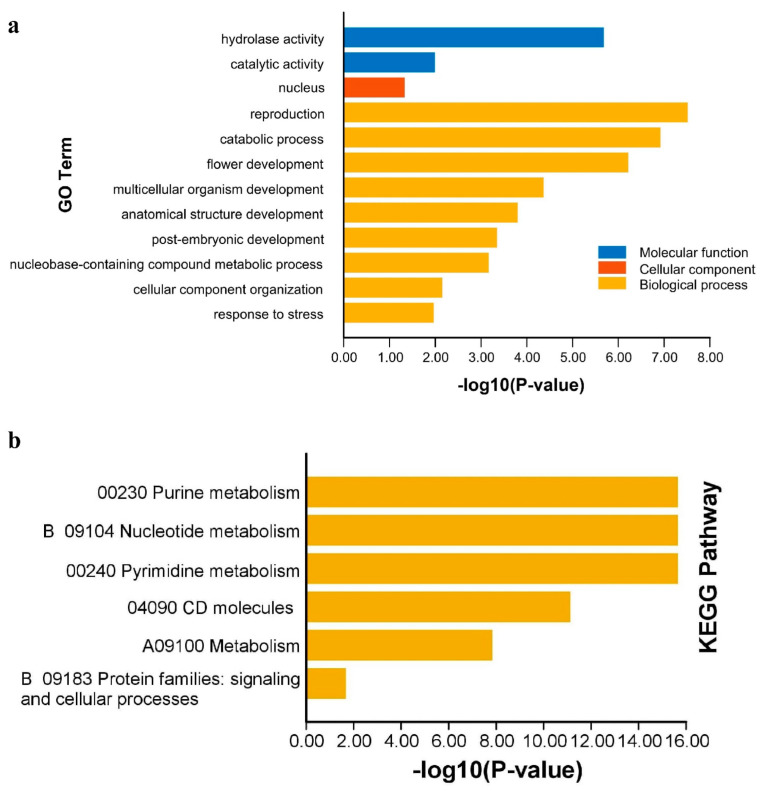
Functional annotation analysis of *AhAPYs*: (**a**) Gene ontology (GO) enrichment analysis. The GO enrichment analysis showed that AhAPYs are enriched in key molecular functions, cellular components, and biological processes. (**b**) KEGG enrichment analysis of *AhAPYs*. The KEGG enrichment analysis depicts the important metabolic roles of *AhAPYs*.

The collinearity analysis was performed among *A. hypogaea*, *A. duranensis*, *A. ipaensis*, and *A. thaliana* to assess their syntenic relationships. Collinearity analysis showed strong evolutionary relationships of *APYs* among these species. *A. hypogaea* showed strong syntenic relationships with its wild parents compared to *Arabidopsis* (Figure 7). The functional annotation analysis was performed to prophesize the potential functions of *AhAPYs*. Gene ontology (GO) enrichment analysis revealed that *AhAPYs* are involved in important GO categories, including molecular functions (MF), cellular components (CC), and biological processes (BP). For the MF category, *AhAPYs* were involved in hydrolase catalytic activity; for the CC category, the *AhGLPs* were mainly found as a component of the nucleus; and for the BP category, *AhAPYs* were involved in a large number of subcategories, including reproduction, catabolic processes, stress responses, and many developmental processes (Figure 8a). These results depict the importance of *AhAPYs* in different metabolic, cellular, and biological processes. The KEGG pathway is a computerized representation of a biological system through which we can infer the role of a protein/gene [49]. We also performed the KEGG enrichment analysis of *AhAPYs* to infer their metabolic roles. *AhAPYs* were involved in key metabolic pathways, including 00230 purine metabolism, B09104 Nucleotide metabolism, 00240 Pyrimidine metabolism, and A09100 metabolism pathways. *AhAPYs* were also enriched in signaling and cellular processes and as 04090 CD molecules (Figure 8b).

### 2.7. Expression Analysis

**Figure 9:**
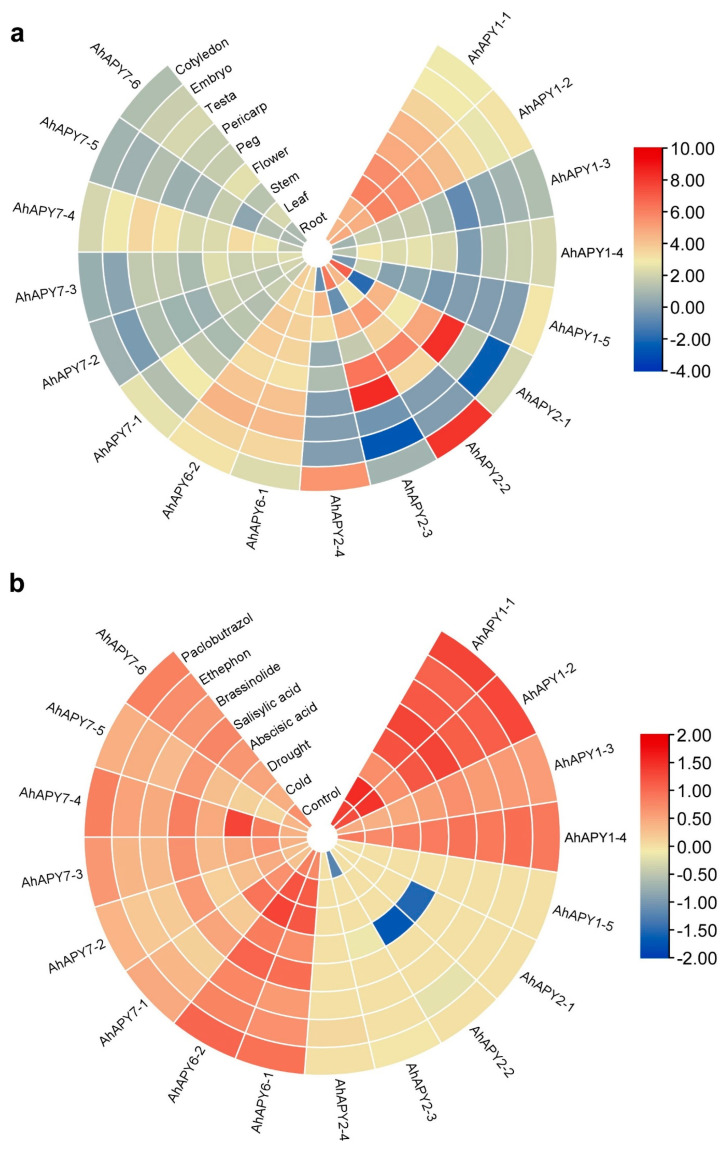
Transcriptome expression of *AhAPYs* in different tissues (log10 normalized values were used to construct the expression heatmap): (**a**) Most AhAPYs did not show any remarkable expression in most tissues. However, some genes showed tissue abundant expression, e.g., *AhAPY2-1* and *AhAPY2-3* showed abundant expression in the pericarp. (**b**) Most of AhAPYs showed constitutively high expression under all treatments (normal and stress), especially *AhAPY1-1* and *AhAPY1-2*.

The transcriptome expression pattern of *AhAPYs* in various tissues and under different hormones/stress treatments was assessed from the peanut genome resource database. All *AhAPYs* showed expression in studied tissues, including leaf, stem, root, flower, peg, pericarp, testa, cotyledons, and embryo. *AhAPY7-1* to *AhAPY7-6* did not show any remarkable expression in the studied tissue. *AhAPY6-1* and *AhAPY6-2* showed uniform expression in all tissues, but expression level was low, and *AhAPY1-1* and *AhAPY1-2* showed relatively higher expression levels in all tissues. *AhAPY2-1* and *AhAPY2-3* showed abundant expression in the pericarp compared to other tissues, and *AhAPY2-2* showed abundant expression in the stem, root, and cotyledons. *AhAPY2-4* showed decreased expression in the stem tip Figure 9a.

*AhAPYs* showed varying expression levels under different hormones and stress treatments. *AhAPY2-1* and *AhAPY2-2* showed decreased expression under ABA treatment. In contrast, *AhAPY1-1* and *AhAPY1-2* showed higher expression under all stress conditions, including ABA, SA, Brassinolide, paclobutrazol, ethephon treatments, drought, normal irrigation, ddH_2_O spray, low temperature, and room temperature. *AhAPY1-5* and *AhAPY2-4* did not respond to the hormones, water, and temperature treatments. The expression matrix of *AhAPYs* in response to different hormones and stress agents is shown in Figure 9b. The FPKM values for transcriptome expression in different tissues and under different hormones are publicly available at (http://peanutgr.fafu.edu.cn/Transcriptome.php; accessed on 10 August 2022).

**Figure 10:**
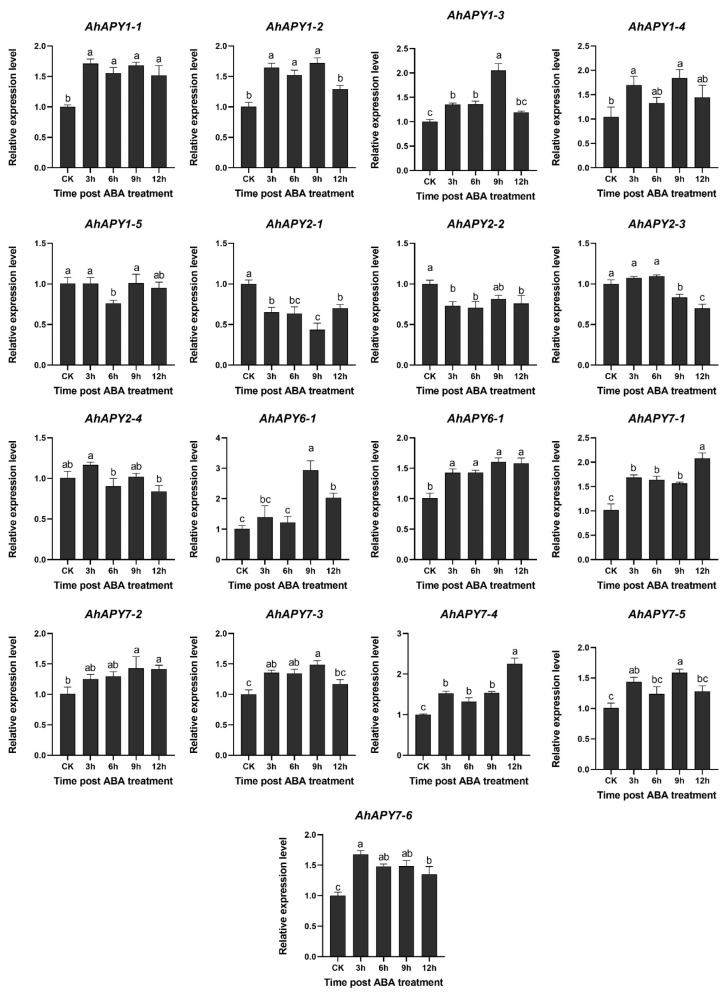
Real-time expression validation of 17 *AhAPY* genes under ABA treatment. The expression of all 17 *APY* genes under the abscisic acid stress was assessed by qRT-PCR analysis. The qRT-PCR results are in accordance with transcriptome data. The data were analyzed by ANOVA, and the significance level was assessed by LSD test at α = 0.05. Different letters (a, b, c etc.,) represents significant differences among expression levels at different time-points.

Further, the expression of all *AhAPYs* genes was assessed under abscisic acid treatment to check whether their expression corresponds to the transcriptome expression. Peanut plants were treated with ABA (10 μg/mL), and quantitative expression was assessed at different time points. The qRT-PCR results validated the transcriptome expression data under ABA treatment. The qRT-PCR-based expression of 17 *AhAPYs* genes is shown in Figure 10. Genes of group 1 (*AhAPY1-1* to *AhAPY1-4*), group 6 (*AhAPY6-*), and group 7 (*AhAPY7-*) showed upregulated transcriptome expression, and the qRT-PCR results also found similar expression pattern. *AhAPY1-5,* and all genes of group 2 (*AhAPY2-*), did not show any change in transcriptome expression, and real-time expression results found a similar expression pattern.

### 2.8. Selection of Gene for Promoter Cloning and In Silico Analysis of Promoter

**Figure 11:**
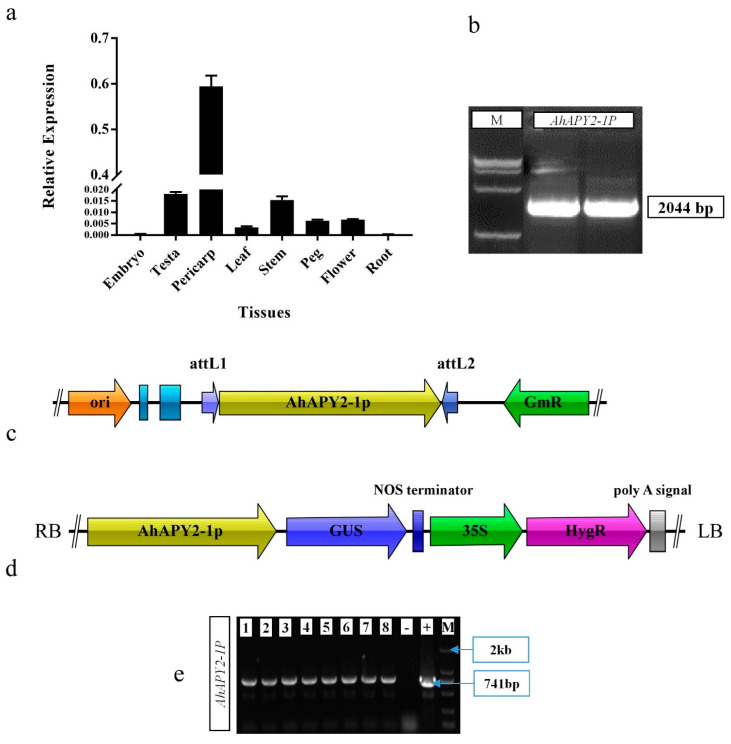
Cloning of *AhAPY2-1* promoter: (**a**) confirmation of *AhAPY2-1* gene expression in different tissues of peanut. *AhAPY2-1* showed high expression in pericarp; (**b**) amplification of *AhAPY2-1* promoter from the DNA template of peanut XHXL cultivar; (**c**) construction of entry vector by gateway BP reaction; (**d**) construction of expression vector by gateway LR reaction; and (**e**) confirmation of T0 transgenic *Arabidopsis* plants. Eight Hygromycin-resistant plants were verified by PCR amplification with promoter-specific forward and GUS gene-specific reverse primer.

In the previous section, the expression profiles showed that the *AhAPY2-1* gene was more highly expressed in the pod or pericarp than in any other tissue; we considered it a pericarp-abundant gene. Based on this consideration, it can be assumed that the promoter of the pericarp-abundant gene can be used to drive a foreign gene in a pod-specific manner. The RNA seq- and gene-chip expression data of *AhAPY2-1* is given in File S1. qPCR was performed to verify the expression of the *AhAPY2-1* gene in different tissues. Results of the qRT-PCR analysis showed a high number of transcripts of *AhAPY2-1* in pod/pericarp tissues compared to all other tissues, indicating its pod-abundant expression pattern Figure 11a. Based on these results, we hypothesized that the promoter of the *AhAPY2-1* gene could be used to drive a foreign gene in a pod-abundant manner.

For cloning of the *AhAPY2-1* promoter, the 2044 bp upstream region was selected and scanned through online promoter analysis databases, the PlantCARE database. It was found that the promoter region contained core promoter elements, including the TATA Box and CAAT box, both of which are required for precise initiation of transcription and tissue-specific activity [50,51]. Aside from these core promoter elements, several other important *cis*-elements were also present in the *AhAPY2-1* promoter. These elements include the hormone-responsive elements auxin (TGA-elements), gibberellin (TATC-box), salicylic acid (TCA-element), abscisic acid (ABRE), methyl jasmonate (TGACG-motif, CGTCA-motif), ethylene responsiveness (ERE), and light-responsive elements (GT1-motif, G-Box, GATA-motif, Box 4, AT-1 motif). Moreover, wound-responsive elements (WUN-motif), defense-related elements (MYB sites), anaerobic induction (ARE), and zein metabolism regulatory element O2-site were also present. Additionally, some elements with unknown functions were also present. Detailed information on the *AhAPY2-1* promoter is provided in Figure S6. The new PLACE database also predicted several key elements in the promoter region, such as seed-specific elements (RY-element) and binding sites for WRKY and MYB transcription factors. Table S4 contains information on *cis*-elements, their position, sequence, and functions predicted by the PLACE database. These elements suggest that the *AhAPY2-1* promoter could be used to replace the native promoter of a gene. The CDS, protein, and promoter sequences of the *AhAPY2-1* gene are given in File S2.

### 2.9. Cloning and Genetic Transformation

The promoter region of the *AhAPY2-1* gene was amplified from the DNA template of the Xinhuixiaoli (XHXL) cultivar with the specific primers (Table S1). Clones having >99% sequence similarity with the original sequence were used to construct the vector. Two-step Gateway cloning was used in which the promoter was first ligated into entry vector pDONR207 by BP reaction. The promoter fragment was ligated into the expression vector pMDC164 by LR reaction in the second step. The complete procedure of amplifying the *AhAPY2-1* promoter and vector construction is shown in Figure 11b–d. Vector pMDC164 contains the Hygromycin resistance gene and the *GUS* reporter gene for the identification of positively transformed plants and functional studies. The expression vector was named *AhAPY2-1P-GUS*.

The expression vector was transformed into *A. tumefaciens*, and (GV3101) cells were used for the genetic transformation of *Arabidopsis* plants by the floral dip method. Positively transformed T0 seeds were screened on Hygromycin (50 mg mL^−1^) selection medium, and eight HygR resistant plants were verified by PCR amplification, Figure 11e. Hygromycin and PCR screening were performed in each generation, and homozygous T3 generation was obtained.

### 2.10. Functional Study of Promoter Activity in Transgenic Plants

**Figure 12:**
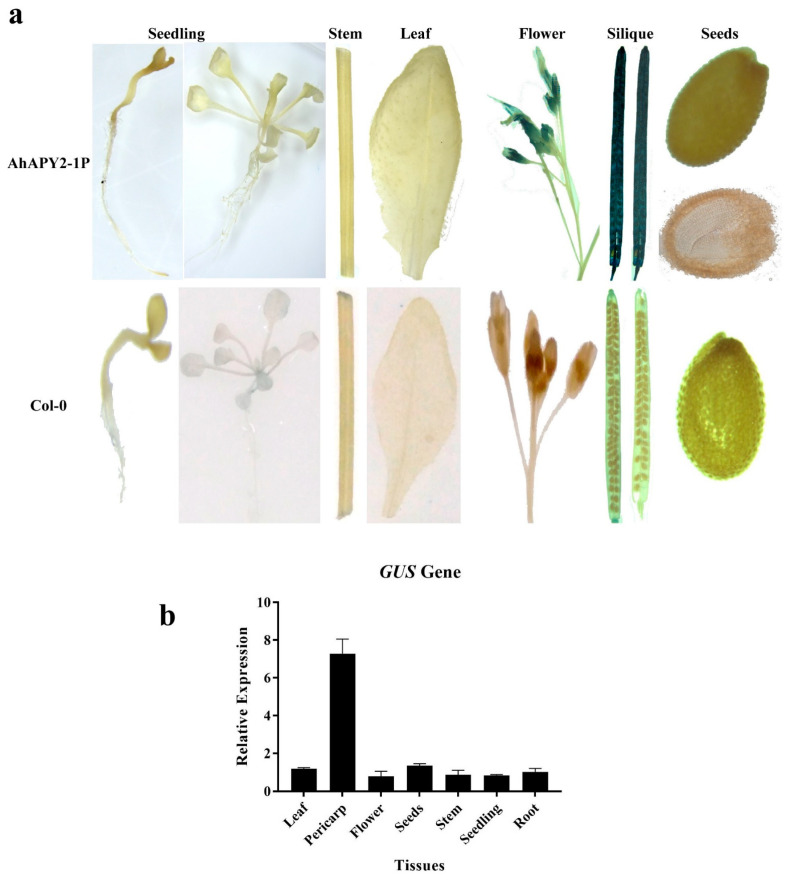
Analysis of *GUS* gene under the control of *AhAPY2-1* promoter in transgenic *Arabidopsis* plants: (**a**) GUS staining of different tissues/organs of transgenic *Arabidopsis* plants. A dense blue color in silique outer coverings/pericarp and also mild staining in flowers. *Arabidopsis* Col-0 plants were used as a control to compare the GUS staining results. (**b**) Quantitative expression of the *GUS* gene under the control of *AhAPY2-1* in transgenic *Arabidopsis* plants. The GUS gene showed relatively high expression in the pericarp of transgenic plants.

Samples from different tissues of transgenic plants were used to check the activity of the *AhAPY2-1*-controlled *GUS* gene. Leaf, roots, stem, seedlings, flowers, siliques, and seeds were incubated in the GUS solution. The GUS staining showed that the siliques outer covering/pod has a dense blue color. Flowers of transgenic *Arabidopsis* plants also showed some blue color after GUS staining, while in all other tissues the GUS staining was very low or absent, Figure 12a. *Arabidopsis* wild plants (Col-0) were also used for GUS staining to compare the results. To assure the proper staining of cotyledons and embryos, seeds were ruptured and incubated in the staining solution. Cryostat sectioning of those seeds was performed to check whether staining was present in cotyledons and embryos. Staining was not detected in any seed tissues, including seed coat/testa, cotyledons, or embryos, Figure 12a. *Arabidopsis* wild type (Col-0) did not show any blue color. The staining results indicated that the *AhAPY2-1* promoter has successfully regulated the GUS gene in a pod/pericarp-abundant manner in transgenic *Arabidopsis* plants.

The qRT-PCR analysis was performed to check the quantitative expression of the *GUS* gene in different tissues of transgenic *Arabidopsis* plants. The qRT-PCR analysis showed that the *GUS* gene was highly expressed in silique outer coverings (pod/pericarp) of transgenic seedlings compared to other tissues, Figure 12b. In contrast, the expression level in all other tissues was very low. Collectively, these results showed that the *AhAPY2-1* promoter effectively drove the expression of the *GUS* gene in the pericarp-abundant manner; hence, this promoter could be a suitable candidate for pod/pericarp-abundant expression of a transgene.

### 2.11. Response of AhAPY2-1 Promoter to Hormones

**Figure 13:**
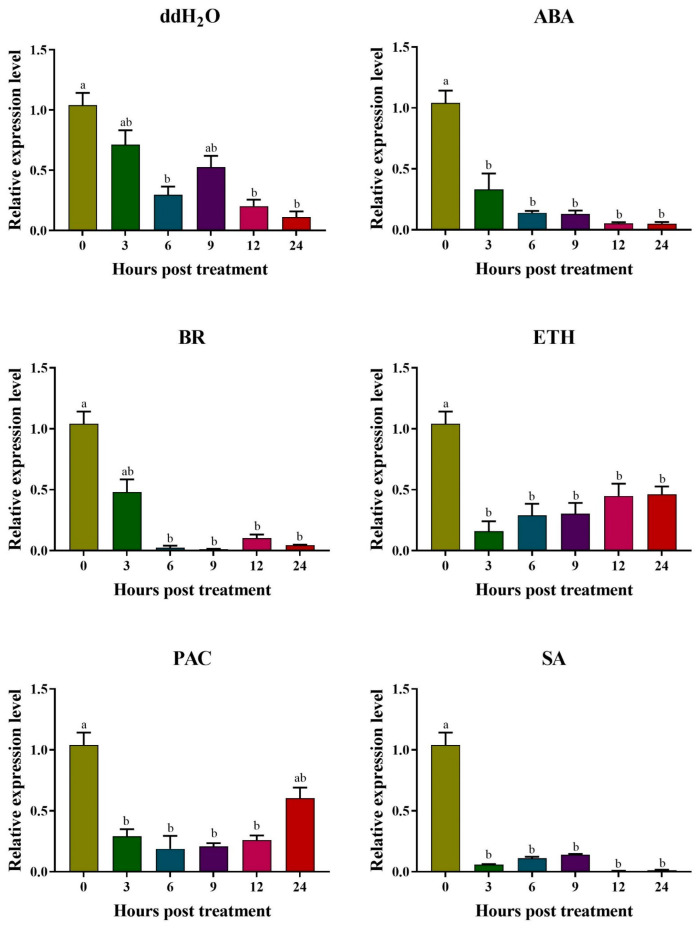
Response of *AhAPY2-1* controlled *GUS* gene to different hormone treatments. The *GUS* gene showed reduced expression in response to different hormones and ddH_2_O spray at all time points. ABA = abscisic acid, BR = Brassinolide, ETH = ethephon, PAC = paclobutrazol, and SA = salicylic acid. Data were analyzed by analysis of variance (ANOVA, *p* < 0.05). Different letters (a, b) represent significant differences among expression levels at different time-points.

Expression responses of *AhAPY2-1* promoter under different phytohormones treatment were studied by exposing the transgenic *Arabidopsis* plants to different hormones stress. The expression of the *GUS* gene controlled by the *AhAPY2-1* promoter was studied to check the activity of the promoter in response to these hormones. The GUS gene showed no remarkable expression under all hormonal/ddH_2_O treatments at all time points. The expression of the *GUS* gene was decreased as compared to the control. Under ABA treatment, expression level decreased at 3 h post-treatment and showed a decreasing trend until 24 h of ABA treatment. Under BR and SA treatment, a similar expression pattern was observed. In response to ethephon and paclobutrazol treatment, the expression level was decreased compared to the control. Still, there was a gradual increase in expression at different time points, but the expression was less than the controlled one. Overall, the expression of the GUS gene was decreased under all treatments than in control plants (Figure 13). These results indicated that the *AhAPY2-1* promoter did not induce *GUS* gene expression under hormone treatments. In other words, we can say that *AhAPY2-1* is a pericarp-specific gene that is not influenced by other stress conditions.

## 3. Discussions

Extracellular ATPs, the energy currency of all organisms, play key regulatory roles in plants and animals. The signaling roles of extracellular ATPs in mammals have been under investigation for the last 40 years [52], but these investigations are at an early stage in plants. Recent studies have demonstrated that extracellular ATP levels play critical roles in regulating various cellular and stress responses [53,54]. Apyrases (APYS)/Nucleoside phosphatases (NTPs) are key regulators of cell growth and stress responses by maintaining extracellular ATP levels [55]. Several studies have shown that cellular ATP levels decrease under stressed conditions, which ultimately results in increased cellular ROS levels causing cell apoptosis [56,57]. Thus, APYs are critical in cleaving the extracellular ATP in the stressed environment to maintain cellular ATP levels and avoid the adverse effects of ROS accumulation. Overexpression of APYs has been reported to inhibit ROS accumulation and increase stress tolerance [36]. During the past decade, some studies have shown that APYs play fundamental roles against cold stress [36], salt stress [58], and drought stress [21]. Their roles in biotic stress tolerance have recently been reported, including powdery mildew response in wheat [59]. These findings provide the future scope of APYs in managing the stress induced by external agents in plants. In *Arabidopsis*, seven apyrases have been reported, and some of them have been functionally characterized [55]. In 2019, Liu and his team investigated apyrases in wheat at a genome-wide scale and reported the defense roles of APYs against powdery mildew [59].

Peanut is an important legume crop worldwide and a livelihood source for many people. At present, peanut is a resource-rich legume with a large amount of data available on yield quality traits [60,61,62,63], diseases/pathogens resistance [5,6,64], seed dormancy [61,65], etc. With the availability of the genome sequence of tetraploid peanut (*Arachis hypogaea*) [10,11], it becomes easy to investigate the apyrases in peanut and study their function. In this study, we used seven *Arabidopsis APYs* and identified 17 *APY* homologs in the cultivated peanut genome (Table 1). These 17 peanut APYs were divided into three phylogenetic groups based on their evolutionary relationships with *Arabidopsis* and soybean. We also identified eight APYs in *each A. duranensis and A. ipaensis* (Table S2). The larger numbers of *APYs* in the genome of cultivated peanut than its diploid parents are due to its tetraploid nature and larger genome size. The evolutionary process has resulted in the structural diversity of genes/gene families in crops; similarly, the APYS of wild and cultivated peanuts possessed large structural diversity. As the number of exons is important in determining gene expression patterns and levels [66], the cultivated peanut also possessed varying exons ranging from 2 to 12 (Figure 2).

The phylogenetic analysis divided the *AhAPYs* into three groups; mainly, these groups correlated to their functions. As previous studies have reported the similar functions of *AtAPY1* and *AtAPY2*, their homologs in peanut tend to cluster in one group. *AtAPY7* homologs in peanuts clustered into a separate group. Although *AtAPY6* and *AtAPY7* possessed almost the same functions, the AtAPY6 homologs clustered into separate groups (Figure 3). A similar phylogenetic grouping has been reported for wheat apyrases [59]. Gene duplication and variations in genome size are key features of genetic diversity. Gene duplication events are important for gene expression diversification and neofunctionalization. We performed the gene duplication analysis for *AhAPYs*. Gene duplication analysis revealed eight duplicated gene pairs (Table 2). Mainly purifying selection pressure was involved in the duplication process, and all of the genes were segmentally duplicated (Figure 4).

Non-coding micro-RNAs are key regulatory elements that play multiple roles in growth, regulation, and defense responses [67]. miRNAs have been a research hotspot for the last few years, and more studies are becoming available on their regulatory roles [47,68,69]. miRNAs are important in managing stress responses. We predicted the putative miRNAs targeting the *AhAPYs* through the published miRNAs data [70]. We found four miRNAs targeting seven *AhAPYs* (Table S3, Figure 5). *Cis*-regulatory elements are of key importance as they determine a gene’s expression pattern, regulatory roles, and stress responses [71,72]. *Cis*-regulatory elements analysis showed important elements involved in light, hormones, growth and development, stress, and defense responses (Figure 6). Genome collinearity and syntenic relations are important evolutionary events that lead to understanding genome duplication and neofunctionalization [73]. The collinearity analysis revealed key syntenic relations of *AhAPYs* with their diploid parents and *Arabidopsis* (Figure 7). Similarly, functional annotation is an important tool to emphasize the functions of a gene/gene family. The gene ontology (GO) analysis showed that AhAPYs are part of molecular functions (MF), Biological processes (BP), and cellular components (CC), and (Figure 8a). The KEGG pathway enrichment analysis showed that *AhAPYs* were also involved in key metabolic pathways (Figure 8b).

Owing to their geocarpic nature, peanut seeds are constantly attacked by soil- and seed-borne diseases. Different fungal, bacterial, and viral pathogens attack peanut plants at different growth stages and deteriorate yield and quality [45,74]. The pod/pericarp is an inedible part of peanut seed and a vital defense organ against various biotic and abiotic stress agents. The promoter is a part of a non-coding DNA sequence present upstream of a gene and regulates the expression of the downstream gene. Previous transgenic projects mainly use constitutive promoters to drive the desired gene(s). The constitutive expression of a foreign gene can have adverse effects on plant growth and development, as the constitutive expression imposes an extra metabolic burden by expressing the gene in tissues/organs where it is not required. Tissue-specific/abundant promoters are suitable alternatives to constitutive promoters as they express the desired gene in a tissue-specific manner. If a tissue-specific promoter with a resistant gene can successfully be transformed into peanut, it can improve the defense capabilities of the plant. We hypothesized that a pericarp-specific promoter would be a good choice to express resistant genes in a pericarp-specific manner to improve the defensive abilities of the pericarp. From the transcriptome expression data (Figure 9), we found that *AhAPY2-1* is abundantly expressed in the pericarp without showing any remarkable expression in other tissues. We selected *AhAPY2-1* for promoter cloning and functional characterization.

Genetic transformation in peanut is a challenging task. There are some reports on successful genetic transformation in peanut [75,76], but not a single well-established protocol exists for transformation. Due to these bottlenecks, *Arabidopsis* becomes a better alternative for the functional characterization of genes and promoters. So, we selected *Arabidopsis* for the functional characterization of the *AhAPY2-1* promoter. We cloned a 2044 bp upstream region of the *AhAPY2-1* gene and analyzed the key *cis*-regulatory elements (Figure S6). *Cis*-regulatory elements clearly showed that *AhAPY2-1P* could be a suitable promoter. The qRT-PCR results validated the transcriptome expression data and clearly showed that this gene expresses in the pericarp-abundant manner (Figure 11a).

The promoter was cloned from peanut variety XHXL, and the plant expression vector was constructed using the backbone of the pMDC164 vector following the gateway cloning system. The floral-dip method was used for the genetic transformation of *Arabidopsis* plants, and positively transformed plants were grown to T3 homozygous generation. GUS staining assay showed strong blue color in the silique’s outer coverings/pericarp; some staining was also present in the flowers. These minute changes in *AhAPY2-1* expression in peanut and *Arabidopsis* are possibly due to the species change. Staining was absent in all other tissues. The cryostat sectioning of dissected seeds showed that the *GUS* gene was not expressed in seed coat/testa, cotyledons, and embryo. Expression of the *GUS* gene determined by qRT-PCR analysis also revealed significantly high expression of the *GUS* gene in the pericarp compared to other tissues. The literature shows that a set of phytohormones plays a key role in regulating several physiological, biochemical, and molecular processes under normal and stressed conditions [77]. Therefore, to check whether the expression of the *GUS* gene under the control of the *AhAPY2-1* promoter in transgenic *Arabidopsis* corresponds to the transcriptome expression of the *AhAPY2-1* gene in response to different hormones or not, transgenic plants were treated with phytohormones, and qPCR was performed. Although there were some deviations in the expression patterns, overall, we can conclude that it followed a similar expression pattern. These findings strongly suggest that *AhAPY2-1P* can regulate a foreign gene’s expression in a pericarp-abundant manner.

## 4. Materials and Methods 

### 4.1. Identification of APYs/NTPs from Peanut Genome

The *APYs* from the peanut genome were identified in a systematic way. First, the protein sequences of seven apyrases from *Arabidopsis AtAPY1*-*AtAPY7* [25] were obtained from the *Arabidopsis* genome database TAIR [78]. The *AtAPYs* protein sequences were used for BLAST search in the PeanutBase database (https://peanutbase.org/pb_sequenceserver; accessed on 2 August 2022) to find the *APYs* in diploid progenitors of cultivated peanut (*A. duranensis* and *A. ipaensis*) [12]. The protein sequences of *AtAPYs*, *AdAPYs*, and *AiAPYs* were used to search the *APYs* in the cultivated peanut genome through BLAST search in Peanut Genome Recourse (PGR) database (http://peanutgr.fafu.edu.cn/; accessed on 2 August 2022) [10]. The presence of the GDA1-CD39 domain (PF01150) in identified *AhAPYs* was confirmed through the NCBI (https://www.ncbi.nlm.nih.gov/; accessed on 2 August 2022) and Pfam databases (http://pfam.xfam.org/; accessed on 2 August 2022).

### 4.2. Genomic Positions, Structure, and Physicochemical Properties of APYs

The genomic positions of *AhAPYs* were determined from the PGR database [10]. Genes were mapped out using the TBtools software [79]. The information regarding the gene structure was also retrieved from the PGR database. The conserved motifs in the protein sequences were elucidated by the MEME suite while setting the maximum number of motifs as ten with other default parameters [80]. Protein tertiary structures were predicted by the ExPASy server, while 3D models were drawn by Swiss-Model (https://www.swissmodel.expasy.org/; accessed on 4 August 2022) following the default parameters. The physicochemical properties, such as theoretical molecular weight (MW) and isoelectric points (pI), were determined by the ProtParam tool (https://web.expasy.org/protparam/; accessed on 3 August 2022) [81], and subcellular localizations were predicted by CELLO v2.5 tool (http://cello.life.nctu.edu.tw/; accessed on 4 August 2022) [82].

### 4.3. Phylogeny and Duplication Analysis

The phylogenetic tree was constructed among the *APYs* of *A. hypogaea*, *G. max*, *A. thaliana*, *A. duranensis*, and *A. ipaensis* to study their evolutionary relationships. MUSCLE program was used to align the protein sequences, and an ML tree with 1000 bootstrap repeats was constructed by MEGA-X. The genome and GFF3 files were run through MCScanX to identify the duplicated gene pairs. A simple Ka/Ks calculator was used to calculate the expected synonymous (Ks) and non-synonymous (Ka) substitution rates. Divergence time (million years ago, MYA) for duplicated gene pairs was calculated as ‘t = Ks/2r’ while using the neutral substitution rate for peanut r = 8.12 × 10^−9^ [12].

### 4.4. Protein–Protein Interaction Analysis and Putative miRNAs Targeting AhAPYs

Possible interactions of *AhAPYs* with other proteins were elucidated by constructing their protein–protein interaction network based on their homologs in *Arabidopsis* using the STRING 11.5 tool (https://www.string-db.org/cgi/; accessed on 5 August 2022). Interacting proteins with 100% similarity and <10^−5^ were considered. The top 10 interactions with a high threshold (0.7) were considered. MCL clustering with inflation parameter 3 was used, and dotted lines were used between cluster edges. The putative miRNAs targeting the peanut *APYs* were predicted through the psRNATarget database (https://www.zhaolab.org/psRNATarget/analysis?function=2; accessed on 7 August 2022) [70]. The CDS sequences of peanut apyrases were scanned through the psRNATarget database for the prediction of putative miRNAs with default settings.

### 4.5. Promoter Cis-Elements Analysis

Online database available for the promoter elements prediction, viz., PlantCARE database (http://bioinformatics.psb.ugent.be/webtools/plantcare/html/; accessed on 7 August 2022) [83] was used to identify the *cis*-elements in APY promoters. The promoter sequences of *AhAPYs* were obtained from the PGR database. For the identification of *cis*-elements, 2 kb upstream sequences were used.

### 4.6. Comparative Genome Synteny and Functional Annotation Analysis

The syntenic relationships of three peanut species and *Arabidopsis* were developed to view the comparative genomic conservations. For this purpose, the genome and GFF3 files of all these species were scanned against each other through One Step MCScanX. The resulting Collinearity, GFF3, and CTL files were merged and used for multiple synteny plots with the help of TBtools [79]. We performed the functional annotation analysis to understand the possible functions/regulatory roles of *AhAPYs*. Whole-genome protein sequences were scanned for functional annotation analysis at the EggNOG server (http://eggnog-mapper.embl.de/; accessed on 8 August 2022). The resulting annotation files were used to perform the GO and KEGG enrichment analysis.

### 4.7. Expression Analysis of AhAPYs

To understand the expression matrices of *AhAPYs* in different tissues and under different hormones/stress agents, the transcriptome expression data were accessed from the peanut genome resource database [10]. The log10 normalized FPKM (fragments per kilobase million) values were used to construct the expression heatmaps for different tissues and under different stress conditions. Further, the transcriptome expression was verified by treating the peanut plants with abscisic acid (ABA) and performing the qRT-PCR analysis. The peanut seeds were grown in small plastic pots, and plants were grown in the greenhouse at 26 °C and 16 h/86 day/night photoperiod. Four-leaf-old plants were treated with ABA (10 μg/mL), and samples were taken at different time points. Total RNA was extracted by the CTAB method with some modification, and the first strand cDNA was synthesized by the PrimeScript 1st strand cDNA Synthesis Kit (Takara, Dalian, China). qRT-PCR analysis was performed to compare the transcriptome and real-time expression of all *APY* genes. The qRT-PCR reaction was performed by the Applied Biosystems 7500 real-time PCR system (Thermofisher Scientific, Waltham, MA, USA) with the cycling program: 94 °C (1 min), 60 °C (1 min), and 72 °C (1 min) for 40 cycles. Peanut *Actin* gene was used as an internal control. Primers used for qRT-PCR are given in Table S1.

### 4.8. Plant Materials and Cloning of AhAPY2-1 Promoter

The seeds of peanut cultivar Xinhuixiaoli (XHXL) and *Arabidopsis* thaliana Col-0, used in this study, were grown in a greenhouse at the Institute of Oil Crops, Fujian Agriculture and Forestry University, Fuzhou, China. Based on the transcriptome expression data, we found that *AhAPY2-1* was abundantly expressed in the pericarp. We selected a 2044 bp upstream region of the *AhAPY2-1* gene for promoter cloning and characterization. The expression of the *AhAPY2-1* gene in different tissues of peanut was checked by the qRT-PCR analyses. The promoter region of the *AhAPY2-1* gene was cloned from the DNA template of peanut variety XHXL, and binary plasmid pMDC164 was used to construct the plant expression vector by the Gateway cloning method. Expression vectors were transformed into *A. tumefaciens* (GV3101) competent cells through the heat shock method. The floral dipping method was used to transform young *Arabidopsis* plants [84]. Positive transgenic plants were identified on a Hygromycin (HygR) selection medium (50 mg mL^−1^), and further verified by PCR amplification. Primers used in this study are given in Table S1. Our previous study gives a detailed procedure for qPCR and promoter cloning and transformation [85]. Eight positive plants (verified by HygR resistance and PCR amplification in every generation) were grown to T3 generation for functional characterization.

### 4.9. Study of Promoter Activity in Transgenic Plants

Different tissues of transgenic *Arabidopsis* plants were used for GUS staining [86] to check the activity of the *GUS* gene under the control of the *AhAPY2-1* promoter. Leaf, stem, flower, seedlings, siliques, and seeds were incubated in GUS staining solution for 12 h and then washed and decolorized with 75% ethanol. To achieve better staining results in embryo and cotyledons, the seeds of transgenic plants were ruptured before incubation. After that, cryostat sectioning was performed with the help of Leica CM1950 Cryostat Microtome (Leica Biosystems, Wetzlar, Germany). The images of stained tissues/organs were taken by Olympus microscope (BX3-CBH) (Olympus, Tokyo, Japan). The quantitative expression of the *GUS* gene in different tissues of transgenic plants was checked by qRT-PCR. Transgenic plants were also treated with different phytohormones; abscisic acid (ABA, 10 μg/mL), Brassinolide (BR, 0.1 mg/L), ethephon (ETH, 1 mg/mL), paclobutrazol (PAC, 150 mg/L), salicylic acid (SA, 3 mmol/L), and low temperature (4 °C). The expression of the *GUS* gene in response to these hormones and low temperature was analyzed by qRT-PCR at different time points.

### 4.10. Statistical Data Analysis

The qRT-PCR was performed for three biological replicates, and data were normalized by the 2^−ΔΔCT^ method. The expression levels were expressed as mean ± standard errors. Statistical significance of expression levels at different time points was assessed by analysis of variance (ANOVA) with significance level α = 0.005 followed by LSD test.

## 5. Conclusions

We investigated the Apyrase (APYs)/Nucleosied phosphatase (NTPs) family in cultivated peanut. Seventeen apyrase homologs were found in the genome of cultivated peanut, while eight homologs were also found in diploid peanut species (*A. duranensis* and *A. ipaensis* each). The genome-wide identification, phylogenetic analysis, structural and expression analysis, GO, and KEGG enrichment, and miRNAs prediction provided a theoretical base for future functional studies. This study highlighted some key genes that effectively respond to different stress agents. *AhAPY1-1, AhAPY1-2, AhAPY6-1,* and *AhAPY6-2* showed upregulated expression against all hormone, water, and temperature treatments. These genes could be suitable candidates for drought and low-temperature resistance. *AhAPY7-4* also showed increased expression against all treatments, while its expression was specifically high against drought treatment. This gene can also be a suitable candidate for drought resistance. Further, we functionally characterized a pericarp/pod-abundant expression promoter. The quantitative PCR analysis validated the transcriptome expression analysis. The quantitative and qualitative expression analysis of the *GUS* gene under *AhAPY2-1P* in transgenic *Arabidopsis* plants provided the practical significance of this promoter in deriving a gene in a pericarp-abundant manner

## Figures and Tables

**Table 1:** Apyrases identified in the genome of cultivated peanut (*Arachis hypogaea* L.).

ID	Renamed	Location	Protein Length (aa)	CDS Length (bp)	Gene Length (bp)	Exons	MW	pI	SC Localization
AH06G22700.1	*AhAPY1-1*	6 (94877757…94883069) -	469	1410	6399	9	51.4852	5.23	Chloroplast
AH16G28170.1	*AhAPY1-2*	16 (122861075…122866396) -	469	1410	6372	9	50.957	5.77	Chloroplast
AH13G57910.1	*AhAPY1-3*	13 (148543072…148553205) +	744	2235	10,134	12	80.6055	6.64	Plasma Membrane
AH10G29400.1	*AhAPY1-4*	10 (111719060…111723323) -	468	1407	4925	9	51.0344	8.05	Chloroplast
AH06G22670.1	*AhAPY1-5*	6 (94715552…94721920) -	133	402	6369	3	15.1463	7.89	Extracellular/Mitochondrial/Nuclear
AH16G28160.1	*AhAPY2-1*	16 (122829171…122837922) -	471	1416	8752	9	52.2322	5.38	Plasma Membrane
AH16G28150.1	*AhAPY2-2*	16 (122697499…122709079) -	464	1395	11,962	9	51.5263	5.18	Plasma Membrane
AH06G22690.1	*AhAPY2-3*	6 (94841125…94849566) -	463	1392	8779	9	51.4852	5.23	Plasma Membrane/Vacuole
AH06G22660.1	*AhAPY2-4*	6 (94712005…94714391) -	133	402	2387	3	14.9691	4.69	Cytoplasmic/Extracellular
AH05G19000.1	*AhAPY6-1*	5 (59386454…59391410) -	571	1716	6212	8	63.0676	9.3	Plasma Membrane
AH15G15910.1	*AhAPY6-2*	15 (61240024…61243866) +	415	1248	4526	6	45.4304	9.69	Plasma Membrane
AH05G01440.1	*AhAPY7-1*	5 (1495293…1498029) +	750	2253	4222	2	82.3927	9.6	Plasma Membrane
AH01G09830.1	*AhAPY7-2*	1 (12576046…12578507) -	719	2160	3829	2	80.8545	9.1	Plasma Membrane
AH20G01300.1	*AhAPY7-3*	20 (1188522…1190990) -	719	2160	3801	2	80.8184	9.1	Plasma Membrane
AH03G20330.1	*AhAPY7-4*	3 (34322499…34325082) +	397	1194	2584	4	43.2395	9.94	Plasma Membrane
AH11G21920.1	*AhAPY7-5*	11 (115305466…115308581) +	519	1560	4173	3	57.6698	7.93	Plasma Membrane
AH01G18780.1	*AhAPY7-6*	1 (75010452…75012336) +	454	1365	4229	2	50.1875	7.6	Plasma Membrane

Note. MW = molecular weight, pI = theoretical isoelectric point, - and + signs represent negative and positive strands.

**Table 2:** Calculation of Ka-Ks and divergence time (million years ago) for duplicated gene pairs.

Seq_1	Seq_2	Ka	Ks	Ka_Ks	Selection Pressure	Time
AhAPY1-1	AhAPY1-3	0.10308	0.76791	0.13424	Purifying	47.28514
AhAPY1-4	AhAPY1-3	0.00954	0.03517	0.27128	Purifying	2.165719
AhAPY1-4	AhAPY2-1	0.32445	1.1245	0.28852	Purifying	69.24287
AhAPY1-5	AhAPY1-4	0.3099	2.06906	0.14978	Purifying	127.405
AhAPY2-4	AhAPY2-2	0.04945	0.07096	0.69685	Purifying	4.36958
AhAPY7-1	AhAPY7-3	0.20883	0.71442	0.2923	Purifying	43.99162
AhAPY7-2	AhAPY7-1	0.20803	0.70387	0.29555	Purifying	43.34174
AhAPY7-2	AhAPY7-3	0.00362	0.02045	0.17706	Purifying	1.259094

Note: Time represents expected divergence time as million years ago (MYA).

## Data Availability

The datasets presented in this study can be found in the online PGR database (http://peanutgr.fafu.edu.cn/; accessed on 2 August 2022). Moreover, the datasets used and/or analyzed during the current study are available from the corresponding author upon reasonable request. However, most of the data is shown in the Supplementary files.

## Supplementary Materials

The following supporting information can be downloaded at: https://www.mdpi.com/article/10.3390/ijms24054622/s1.

## References

[1] Varshney R.K., Pandey M.K., Puppala N. (2017). The Peanut Genome: An Introduction. The Peanut Genome.

[2] FAOSTAT (2020). Food and Agriculture Organization of the United Nations (FAO). https://fenix.fao.org/faostat/internal/en/#data/QCL/visualize.

[3] Kumar R., Pandey M.K., Roychoudhry S., Nayyar H., Kepinski S., Varshney R.K. (2019). Peg biology: Deciphering the molecular regulations involved during peanut peg development. Front. Plant Sci..

[4] Haro R.J., Mantese A., Otegui M.E. (2011). Peg viability and pod set in peanut: Response to impaired pegging and water deficit. Flora.

[5] Soni P., Gangurde S.S., Ortega-Beltran A., Kumar R., Parmar S., Sudini H.K., Lei Y., Ni X., Huai D., Fountain J.C. (2020). Functional biology and molecular mechanisms of host-pathogen interactions for aflatoxin contamination in groundnut (*Arachis hypogaea* L.) and maize (*Zea mays* L.). Front. Microbiol..

[6] Pandey M.K., Kumar R., Pandey A.K., Soni P., Gangurde S.S., Sudini H.K., Fountain J.C., Liao B., Desmae H., Okori P. (2019). Mitigating aflatoxin contamination in groundnut through a combination of genetic resistance and post-harvest management practices. Toxins.

[7] Xie M., Chung C.Y.-L., Li M.-W., Wong F.-L., Wang X., Liu A., Wang Z., Leung A.K.-Y., Wong T.-H., Tong S.-W. (2019). A reference-grade wild soybean genome. Nat. Commun..

[8] Zhang J., Zhang X., Tang H., Zhang Q., Hua X., Ma X., Zhu F., Jones T., Zhu X., Bowers J. (2018). Allele-defined genome of the autopolyploid sugarcane *Saccharum spontaneum* L. Nat. Genet..

[9] Schmutz J., McClean P.E., Mamidi S., Wu G.A., Cannon S.B., Grimwood J., Jenkins J., Shu S., Song Q., Chavarro C. (2014). A reference genome for common bean and genome-wide analysis of dual domestications. Nat. Genet..

[10] Zhuang W., Chen H., Yang M., Wang J., Pandey M.K., Zhang C., Chang W.-C., Zhang L., Zhang X., Tang R. (2019). The genome of cultivated peanut provides insight into legume karyotypes, polyploid evolution and crop domestication. Nat. Genet..

[11] Bertioli D.J., Jenkins J., Clevenger J., Dudchenko O., Gao D., Seijo G., Leal-Bertioli S., Ren L., Farmer A.D., Pandey M.K. (2019). The genome sequence of segmental allotetraploid peanut Arachis hypogaea. Nat. Genet..

[12] Bertioli D.J., Cannon S.B., Froenicke L., Huang G., Farmer A.D., Cannon E.K., Liu X., Gao D., Clevenger J., Dash S. (2016). The genome sequences of Arachis duranensis and Arachis ipaensis, the diploid ancestors of cultivated peanut. Nat. Genet..

[13] Song H., Wang P., Lin J.-Y., Zhao C., Bi Y., Wang X. (2016). Genome-wide identification and characterization of WRKY gene family in peanut. Front. Plant Sci..

[14] Gao C., Sun J., Wang C., Dong Y., Xiao S., Wang X., Jiao Z.J.P.O. (2017). Genome-wide analysis of basic/helix-loop-helix gene family in peanut and assessment of its roles in pod development. PLoS ONE.

[15] Wang Z., Yan L., Wan L., Huai D., Kang Y., Shi L., Jiang H., Lei Y., Liao B. (2019). Genome-wide systematic characterization of bZIP transcription factors and their expression profiles during seed development and in response to salt stress in peanut. BMC Genom..

[16] Zhao K., Li K., Ning L., He J., Ma X., Li Z., Zhang X., Yin D. (2019). Genome-wide analysis of the growth-regulating factor family in peanut (*Arachis hypogaea* L.). Int. J. Mol. Sci..

[17] Tang Y., Du G., Xiang J., Hu C., Li X., Wang W., Zhu H., Qiao L., Zhao C., Wang J. (2022). Genome-wide identification of auxin response factor (ARF) gene family and the miR160-ARF18-mediated response to salt stress in peanut (*Arachis hypogaea* L.). Genomics.

[18] Raza A., Sharif Y., Chen K., Wang L., Fu H., Zhuang Y., Chitikineni A., Chen H., Zhang C., Varshney R.K. (2022). Genome-wide characterization of ascorbate peroxidase (APX) gene family in peanut (*Arachis hypogea* L.) revealed their crucial role in growth and multiple stress tolerance. Front. Plant Sci..

[19] Tang Y., Qin S., Guo Y., Chen Y., Wu P., Chen Y., Li M., Jiang H., Wu G. (2016). Genome-wide analysis of the AP2/ERF gene family in physic nut and overexpression of the JcERF011 gene in rice increased its sensitivity to salinity stress. PLoS ONE.

[20] Cao Y., Tanaka K., Nguyen C.T., Stacey G. (2014). Extracellular ATP is a central signaling molecule in plant stress responses. Curr. Opin. Plant Biol..

[21] Veerappa R., Slocum R.D., Siegenthaler A., Wang J., Clark G., Roux S. (2019). Ectopic expression of a pea apyrase enhances root system architecture and drought survival in Arabidopsis and soybean. Plant Cell Environ..

[22] Masoud H.M., Helmy M.S., Darwish D.A., Abdel-Monsef M.M., Ibrahim M.A. (2020). Apyrase with anti-platelet aggregation activity from the nymph of the camel tick *Hyalomma dromedarii*. Exp. Appl. Acarol..

[23] Proietti M., Perruzza L., Scribano D., Pellegrini G., D’Antuono R., Strati F., Raffaelli M., Gonzalez S.F., Thelen M., Hardt W.-D. (2019). ATP released by intestinal bacteria limits the generation of protective IgA against enteropathogens. Nat. Commun..

[24] Da’dara A.A., Bhardwaj R., Ali Y.B., Skelly P.J. (2014). Schistosome tegumental ecto-apyrase (SmATPDase1) degrades exogenous pro-inflammatory and pro-thrombotic nucleotides. PeerJ.

[25] Chiu T.-Y., Lao J., Manalansan B., Loqué D., Roux S.J., Heazlewood J.L. (2015). Biochemical characterization of Arabidopsis APYRASE family reveals their roles in regulating endomembrane NDP/NMP homoeostasis. Biochem. J..

[26] Dunkley T.P., Watson R., Griffin J.L., Dupree P., Lilley K.S. (2004). Localization of organelle proteins by isotope tagging (LOPIT). Mol. Cell. Proteom..

[27] Leal D.B., Streher C.A., Neu T.N., Bittencourt F.P., Leal C.A., da Silva J.E., Morsch V.M., Schetinger M.R. (2005). Characterization of NTPDase (NTPDase1; ecto-apyrase; ecto-diphosphohydrolase; CD39; EC 3.6. 1.5) activity in human lymphocytes. Biochim. Et Biophys. Acta.

[28] Yang J. (2011). Functional Analyses of Arabidopsis Apyrases 3 through 7.

[29] Raza A., Tabassum J., Fakhar A.Z., Sharif R., Chen H., Zhang C., Ju L., Fotopoulos V., Siddique K.H., Singh R.K. (2022). Smart reprograming of plants against salinity stress using modern biotechnological tools. Crit. Rev. Biotechnol..

[30] Sun J., Zhang C., Zhang X., Deng S., Zhao R., Shen X., Chen S. (2012). Extracellular ATP signaling and homeostasis in plant cells. Plant Signal. Behav..

[31] Clark G., Fraley D., Steinebrunner I., Cervantes A., Onyirimba J., Liu A., Torres J., Tang W., Kim J., Roux S.J. (2011). Extracellular nucleotides and apyrases regulate stomatal aperture in Arabidopsis. Plant Physiol..

[32] Yang X., Wang B., Farris B., Clark G., Roux S.J. (2015). Modulation of root skewing in Arabidopsis by apyrases and extracellular ATP. Plant Cell Physiol..

[33] Lim M.H., Wu J., Yao J., Gallardo I.F., Dugger J.W., Webb L.J., Huang J., Salmi M.L., Song J., Clark G. (2014). Apyrase suppression raises extracellular ATP levels and induces gene expression and cell wall changes characteristic of stress responses. Plant Physiol..

[34] Yang J., Wu J., Romanovicz D., Clark G., Roux S.J.J.P.p., biochemistry (2013). Co-regulation of exine wall patterning, pollen fertility and anther dehiscence by Arabidopsis apyrases 6 and 7. Plant Physiol. Biochem..

[35] Shiraishi T. (2013). Suppression of defense response related to plant cell wall. Jpn. Agric. Res. Q..

[36] Deng S., Sun J., Zhao R., Ding M., Zhang Y., Sun Y., Wang W., Tan Y., Liu D., Ma X. (2015). Populus euphratica APYRASE2 enhances cold tolerance by modulating vesicular trafficking and extracellular ATP in Arabidopsis plants. Plant Physiol..

[37] Sun J., Zhang X., Deng S., Zhang C., Wang M., Ding M., Zhao R., Shen X., Zhou X., Lu C.J.P.o. (2012). Extracellular ATP signaling is mediated by H_2_O_2_ and cytosolic Ca^2+^ in the salt response of Populus euphratica cells. PLoS ONE.

[38] Alam I., Lee D.-G., Kim K.-H., Park C.-H., Sharmin S.A., Lee H., Oh K.-W., Yun B.-W., Lee B.-H. (2010). Proteome analysis of soybean roots under waterlogging stress at an early vegetative stage. J. Biosci..

[39] Rajesh M.K., Gangurde S.S., Pandey M.K., Niral V., Sudha R., Jerard B.A., Kadke G.N., Sabana A.A., Muralikrishna K.S., Samsudeen K. (2021). Insights on Genetic Diversity, Population Structure, and Linkage Disequilibrium in Globally Diverse Coconut Accessions Using Genotyping-by-Sequencing. OMICS A J. Integr. Biol..

[40] Jadhav M.P., Gangurde S.S., Hake A.A., Yadawad A., Mahadevaiah S.S., Pattanashetti S.K., Gowda M., Shirasawa K., Varshney R.K., Pandey M.K. (2021). Genotyping-by-Sequencing Based Genetic Mapping Identified Major and Consistent Genomic Regions for Productivity and Quality Traits in Peanut. Front. Plant Sci..

[41] Hong Y., Pandey M.K., Lu Q., Liu H., Gangurde S.S., Li S., Liu H., Li H., Liang X., Varshney R.K. (2021). Genetic diversity and distinctness based on morphological and SSR markers in peanut. Agron. J..

[42] Gangurde S.S., Nayak S.N., Joshi P., Purohit S., Sudini H.K., Chitikineni A., Hong Y., Guo B., Chen X., Pandey M.K. (2021). Comparative transcriptome analysis identified candidate genes for late leaf spot resistance and cause of defoliation in groundnut. Int. J. Mol. Sci..

[43] Sharma M., Gangurde S.S., Salgotra R.K., Kumar B., Singh A.K., Pandey M.K. (2021). Genetic mapping for grain quality and yield-attributed traits in Basmati rice using SSR-based genetic map. J. Biosci..

[44] Pandey M.K., Gangurde S.S., Sharma V., Pattanashetti S.K., Naidu G.K., Faye I., Hamidou F., Desmae H., Kane N.A., Yuan M. (2020). Improved genetic map identified major QTLs for drought tolerance-and iron deficiency tolerance-related traits in groundnut. Genes.

[45] Khan S.A., Chen H., Deng Y., Chen Y., Zhang C., Cai T., Ali N., Mamadou G., Xie D., Guo B. (2020). High-density SNP map facilitates fine mapping of QTLs and candidate genes discovery for Aspergillus flavus resistance in peanut (*Arachis hypogaea*). Theor. Appl. Genet..

[46] Yang Z., Bielawski J.P. (2000). Statistical methods for detecting molecular adaptation. Trends Ecol. Evol..

[47] Ding Y., Ma Y., Liu N., Xu J., Hu Q., Li Y., Wu Y., Xie S., Zhu L., Min L. (2017). microRNAs involved in auxin signalling modulate male sterility under high-temperature stress in cotton (*Gossypium hirsutum*). Plant J..

[48] Sun X., Xu L., Wang Y., Yu R., Zhu X., Luo X., Gong Y., Wang R., Limera C., Zhang K. (2015). Identification of novel and salt-responsive miRNAs to explore miRNA-mediated regulatory network of salt stress response in radish (*Raphanus sativus* L.). BMC Genom..

[49] Kanehisa M., Goto S., Hattori M., Aoki-Kinoshita K.F., Itoh M., Kawashima S., Katayama T., Araki M., Hirakawa M. (2006). From genomics to chemical genomics: New developments in KEGG. Nucleic Acids Res..

[50] Grace M.L., Chandrasekharan M.B., Hall T.C., Crowe A.J. (2004). Sequence and spacing of TATA box elements are critical for accurate initiation from the β-phaseolin promoter. J. Biol. Chem..

[51] Shirsat A., Wilford N., Croy R., Boulter D.J.M., MGG G.G. (1989). Sequences responsible for the tissue specific promoter activity of a pea legumin gene in tobacco. Mol. Gen. Genet..

[52] Choi J., Tanaka K., Liang Y., Cao Y., Lee S.Y., Stacey G. (2014). Extracellular ATP, a danger signal, is recognized by DORN1 in Arabidopsis. Biochem. J..

[53] Roux S.J., Steinebrunner I. (2007). Extracellular ATP: An unexpected role as a signaler in plants. Trends Plant Sci..

[54] Tanaka K., Gilroy S., Jones A.M., Stacey G. (2010). Extracellular ATP signaling in plants. Trends Cell Biol..

[55] Clark G.B., Morgan R.O., Fernandez M.-P., Salmi M.L., Roux S.J. (2014). Breakthroughs spotlighting roles for extracellular nucleotides and apyrases in stress responses and growth and development. Plant Sci..

[56] Song C.J., Steinebrunner I., Wang X., Stout S.C., Roux S. (2006). Extracellular ATP induces the accumulation of superoxide via NADPH oxidases in Arabidopsis. Plant Physiol..

[57] Demidchik V., Shang Z., Shin R., Thompson E., Rubio L., Laohavisit A., Mortimer J.C., Chivasa S., Slabas A.R., Glover B. (2009). Plant extracellular ATP signalling by plasma membrane NADPH oxidase and Ca^2+^ channels. Plant J..

[58] Liu D., Deng S., Zhang Y., Zhang X., Sun J., Wang M., Zhao R., Jing Y., Shen X., Chen S. (2013). Cloning of apyrase gene PeAPY2 from Populus euphratica and the salt tolerance of the transformed cells. Genom. Appl. Biol..

[59] Liu W., Ni J., Shah F.A., Ye K., Hu H., Wang Q., Wang D., Yao Y., Huang S., Hou J. (2019). Genome-wide identification, characterization and expression pattern analysis of APYRASE family members in response to abiotic and biotic stresses in wheat. PeerJ.

[60] Gangurde S.S., Wang H., Yaduru S., Pandey M.K., Fountain J.C., Chu Y., Isleib T., Holbrook C.C., Xavier A., Culbreath A.K. (2020). Nested-association mapping (NAM)-based genetic dissection uncovers candidate genes for seed and pod weights in peanut (Arachis hypogaea). Plant Biotechnol. J..

[61] Kumar R., Janila P., Vishwakarma M.K., Khan A.W., Manohar S.S., Gangurde S.S., Variath M.T., Shasidhar Y., Pandey M.K., Varshney R.K. (2020). Whole-genome resequencing-based QTL-seq identified candidate genes and molecular markers for fresh seed dormancy in groundnut. Plant Biotechnol. J..

[62] Tayade A.D., Motagi B.N., Jadhav M.P., Nadaf A.S., Koti R.V., Gangurde S.S., Sharma V., Varshney R.K., Pandey M.K., Bhat R.S. (2022). Genetic mapping of tolerance to iron deficiency chlorosis in peanut (*Arachis hypogaea* L.). Euphytica.

[63] Zhang K., Ma J., Gangurde S.S., Hou L., Xia H., Li N., Pan J., Tian R., Huang H., Wang X. (2022). Targeted metabolome analysis reveals accumulation of metabolites in testa of four peanut germplasms. Front. Plant Sci..

[64] Shasidhar Y., Variath M., Vishwakarma M., Manohar S., Gangurde S., Sriswathi M., Sudini H., Dobariya K., Bera S., Radhakrishnan T. (2020). Improvement of three Indian popular groundnut varieties for foliar disease resistance and high oleic acid using SSR markers and SNP array in marker-assisted backcrossing. Crop J..

[65] Bomireddy D., Gangurde S.S., Variath M.T., Janila P., Manohar S.S., Sharma V., Parmar S., Deshmukh D., Reddisekhar M., Reddy D.M. (2022). Discovery of Major Quantitative Trait Loci and Candidate Genes for Fresh Seed Dormancy in Groundnut. Agronomy.

[66] Guo Y., Qiu L.-J. (2013). Genome-wide analysis of the Dof transcription factor gene family reveals soybean-specific duplicable and functional characteristics. PLoS ONE.

[67] Su W., Raza A., Gao A., Jia Z., Zhang Y., Hussain M.A., Mehmood S.S., Cheng Y., Lv Y., Zou X. (2021). Genome-Wide Analysis and Expression Profile of Superoxide Dismutase (SOD) Gene Family in Rapeseed (*Brassica napus* L.) under Different Hormones and Abiotic Stress Conditions. Antioxidants.

[68] Chen H., Yang Q., Chen K., Zhao S., Zhang C., Pan R., Cai T., Deng Y., Wang X., Chen Y. (2019). Integrated microRNA and transcriptome profiling reveals a miRNA-mediated regulatory network of embryo abortion under calcium deficiency in peanut (*Arachis hypogaea* L.). BMC Genom..

[69] Xie F., Zhang B. (2015). micro RNA evolution and expression analysis in polyploidized cotton genome. Plant Biotechnol. J..

[70] Dai X., Zhuang Z., Zhao P.X. (2018). psRNATarget: A plant small RNA target analysis server (2017 release). Nucleic Acids Res..

[71] Maruyama-Nakashita A., Nakamura Y., Watanabe-Takahashi A., Inoue E., Yamaya T., Takahashi H. (2005). Identification of a novel cis-acting element conferring sulfur deficiency response in Arabidopsis roots. Plant J..

[72] Osakabe Y., Yamaguchi-Shinozaki K., Shinozaki K., Tran L.S.P. (2014). ABA control of plant macroelement membrane transport systems in response to water deficit and high salinity. New Phytol..

[73] Chen Y., Song W., Xie X., Wang Z., Guan P., Peng H., Jiao Y., Ni Z., Sun Q., Guo W. (2020). A collinearity-incorporating homology inference strategy for connecting emerging assemblies in the triticeae tribe as a pilot practice in the plant pangenomic era. Mol. Plant.

[74] Zhang C., Chen H., Cai T., Deng Y., Zhuang R., Zhang N., Zeng Y., Zheng Y., Tang R., Pan R. (2017). Overexpression of a novel peanut NBS-LRR gene A h RRS 5 enhances disease resistance to Ralstonia solanacearum in tobacco. Plant Biotechnol. J..

[75] Karthik S., Pavan G., Sathish S., Siva R., Kumar P.S., Manickavasagam M. (2018). Genotype-independent and enhanced in planta Agrobacterium tumefaciens-mediated genetic transformation of peanut [*Arachis hypogaea* (L.)]. 3 Biotech.

[76] Wei H.H., Yu S.T., Wang Z.W., Yang Z., Song G.S., Wang X.Z., Sun X.S., Wang C.T. (2021). In Planta Genetic Transformation to Produce CRISPRed High-Oleic Peanut. Prepr. Res. Sq..

[77] Raza A., Salehi H., Rahman M.A., Zahid Z., Haghjou M.M., Najafi-Kakavand S., Charagh S., Osman H.S., Albaqami M., Zhuang Y. (2022). Plant hormones and neurotransmitter interactions mediate antioxidant defenses under induced oxidative stress in plants. Front. Plant Sci..

[78] Lamesch P., Berardini T.Z., Li D., Swarbreck D., Wilks C., Sasidharan R., Muller R., Dreher K., Alexander D.L., Garcia-Hernandez M. (2012). The Arabidopsis Information Resource (TAIR): Improved gene annotation and new tools. Nucleic Acids Res..

[79] Chen C., Chen H., Zhang Y., Thomas H.R., Frank M.H., He Y., Xia R. (2020). TBtools: An integrative toolkit developed for interactive analyses of big biological data. Mol. Plant.

[80] Bailey T.L., Johnson J., Grant C.E., Noble W.S. (2015). The MEME suite. Nucleic Acids Res..

[81] Gasteiger E., Gattiker A., Hoogland C., Ivanyi I., Appel R.D., Bairoch A.J.N.a.r. (2003). ExPASy: The proteomics server for in-depth protein knowledge and analysis. Nucleic Acids Res..

[82] Yu C.S., Chen Y.C., Lu C.H., Hwang J.K. (2006). Prediction of protein subcellular localization. Proteins Struct. Funct. Bioinform..

[83] Lescot M., Déhais P., Thijs G., Marchal K., Moreau Y., Van de Peer Y., Rouzé P., Rombauts S. (2002). PlantCARE, a database of plant cis-acting regulatory elements and a portal to tools for in silico analysis of promoter sequences. Nucleic Acids Res..

[84] Clough S.J., Bent A.F. (1998). Floral dip: A simplified method for Agrobacterium-mediated transformation of Arabidopsis thaliana. Plant J..

[85] Sharif Y., Chen H., Deng Y., Ali N., Khan S., Zhang C., Xie W., Chen K., Cai T., Yang Q. (2022). Cloning and Functional Characterization of a Pericarp Abundant Expression Promoter (AhGLP17-1P) From Peanut (*Arachis hypogaea* L.). Front. Genet..

[86] Jefferson R.A., Kavanagh T.A., Bevan M.W. (1987). GUS fusions: Beta-glucuronidase as a sensitive and versatile gene fusion marker in higher plants. EMBO J..

