# Supplementary material for: Genome-Wide Investigation of Apyrase (APY) Genes in Peanut (Arachis hypogaea L.) and Functional Characterization of a Pod-Abundant Expression Promoter AhAPY2-1p
Source: Int J Mol Sci. 2023 Feb 27;24(5):4622. doi: 10.3390/ijms24054622 (PMC10003104; doi:10.3390/ijms24054622)
Supplement: Supplementary file 1 [file ijms-24-04622-s001.zip › File S2.pdf]

File S1: CDS, Protein, and promoter sequences of *AhAPY2-1* gene**.**

Peanut Gene ID	AH16G28160
Peanut Transcript ID	AH16G28160.1
Description	Nucleoside-triphosphatase/apyrase 2 (GDA1/CD39)

Sequences

CDS

>AH16G28160.1

ATGCAAGTGCAAATTGAAAAGATGGATCTCAAAAGCCTCTTCACATTTTTACTCTTATTGATG
CCATCATTCTGTTCCTCTCAATCCCTGACGATTAACAACATTTTCTTTACTCATCGTAAGATAC
TTCCAAAACAAAAATCCAATGATAATAATAACAATAACGATGTTACTTCTTATGCTGTCATCT
TTGATGCTGGTAGCTCCGGATCTCGTGTTCATGTCTTCCATTTTGATCAGAATCTCAACCTTCTT
CATATTGGCAACGAAATTGAGTTTTATAAGAAGATCGAACCCGGTTTGAGTGCATATGCAGAT
AATCCAGAAGAAGCAGCAGAATCTCTGATTCCACTCTTAGACGAAGCACAGGATATTATTCCA
CCGCAACTCCAACCCAAAACACCCCTTAGGCTTGGGGCAACTGCAGGTTTAAGGTTGTTGGAG
GGTGATACTGCTGAAAGAATATTGCAAGCGGTTAGGGATATGTTCAGGACAAGAAGTAACCT
GAACGTTCAATCTGATGCAGTAACTGTTATTGATGGAACCCAAGAAGGTTCTTATATGTGGGT
GGCACTAAACTATCTACTGGGAAACTTGGGAAACAAGTATTCAAGGACGGTGGGAGTGGTTG
ATCTTGGAGGTGCATCAGTTCAAATGGCATATGCTATTTCAAAGCACGCAGCTCAAAATGCAC
CATTACCACCAGATGGACAGGACCCATACATTAAAAATGTCATTCTTAAAGGAACCGAATATA
ACCTCTATGTTCATAGTTACTTGCGCTATGGTAGTGATGCTGCTCGTGCTGAAATTTTGAACAT
CACTCATAATTCTGCTAATCCTTGCGTTTTAGCTGACTTTGATGGGACATATGCATACTCAGGA
AATGAGTATAGGGCATATGCACCAAGTTCAGGGTCCAGCTTTGAAAAATGCAGGCATATAGT
GCGTAAGGTCCTCAAATTGGACGCACCATGTCCATATAATAAAGATTGCACTTTTGGTGGAAT
ATGGAGTGGTGGAGGTGGTAATGGACAAAAAAATCTCTATGTTGCTACGTCTTTCTTTTATAT
GGCATCACAGACGGGTATAGTTGATCCGAACAAACCCAATTCGAAGGTTATTCCGGCGGATTA
CAAGGTTGCAGCTAAGAAAGCATGTAATACAAAATATGAGGATGCTGCATCTATTTATCCTCT
TATTACCATTGACAGACTTCCTTATGTCTGCATGGATCTCACATACCAATATACATTGCTCGTT
GAGGGATTCGGCATAGAACCATGGCAAGAGATTACAGTCACTCAGCAAATTGAATATGAAGA
TGCTTCCGTAGATGCAAGCTGGCCTCTAGGGAGTGCCATAGAAGCCATATCTTCATTACCTAA
ATTTGAGCGATTGATGTATTTTATTTAA

Protein

>AH16G28160.1

MQVQIEKMDLKSLFTFLLLLMPSFCSSQSLTINNIFFTHRKILPKQKSNDNNNNNDVTSYAVIFDAG
SSGSRVHVFHFDQNLNLLHIGNEIEFYKKIEPGLSAYADNPEEAAESLIPLLDEAQDIIPPQLQPKTPL
RLGATAGLRLLEGDTAERILQAVRDMFRTRSNLNVQSDAVTVIDGTQEGSYMWVALNYLLGNLG
NKYSRTVGVVDLGGASVQMAYAISKHAAQNAPLPPDGQDPYIKNVILKGTEYNLYVHSYLRYGS
DAARAEILNITHNSANPCVLADFDGTYAYSGNEYRAYAPSSGSSFEKCRHIVRKVLKLDAPCPYNK
DCTFGGIWSGGGGNGQKNLYVATSFFYMASQTGIVDPNKPNSKVIPADYKVAAKKACNTKYEDA
ASIYPLITIDRLPYVCMDLTYQYTLLVEGFGIEPWQEITVTQQIEYEDASVDASWPLGSAIEAISSLP
KFERLMYFI

Promoter

> AH16G28160.1

TGACGATAATTGTAATGGTCTGCGAATTATAAGATGAACTTTGTAATGGTCTGCGAGACAACT
TTTACATGATGCAAAAGTGGAGCTAGAGGCGCGTCTAAAGAGGTAGGGAACATGTGATATAT
CCTGAAAATGACGTGAGAATTATTTTAAGCGGACGATATATATCCCAAACTAGATACAATTAT
TTTCAATAAATTAATCTTTTGATATACAATATTCGTGAAAATGAATTGTGTTTTTATTCATCAT
ACATAAGATCTGTTTGTTAAATAAAATAAAATTGTTTTTTAATAAACATAAGTCATACAAATA
TTATTTGATAAAATTATTCTTAAAATTTAAAATTGATTAATAGTGAAATTCGTTCTTTAAAAAT
AATTTATTTTTTAAATTAGTTTTTAAAAAATTAAATTAGTCATATTAGTTCTTAAAAGATAAGA
TATAAGTCAAATTGGCCTTTCTATTAGTCAGATAATGACGTGTCATATGAAATGCCACGTGTC
ATGATGACGTGGTAGATTAATACCACGTAACATAAGATGATTAGCCGATGTGTATCACGTATT
ATCTAATATGTCATAAATTTATTTAAGTCAAATTAATTCTAAAAGTACACACGCAAATCATTTT
CATTATTAAAATTTTAAAAATTAATCAAATTAGTTCTTATATAAATCTCTTTTATTTTTTTTATA
ATATTAAATTTTAGATAAATTCTACTATACTCTTTTTAAAGTGACATGTAATTGTAAATTACTC
TGAGTGATCATTTTATGATCGAATACTATCTTAATCATAATTAGGCCATTTTATAATTAAAAAA
TTATTTGAAAAGGGTGAATGTATAATTTAATAAAATATTAAAAATTGAATCCTATTTTTTTTTT
CAAATCAATCTTTTAAAAAATGTATCCTAACTCTCTTTAATGGTTGTCGCTCCTTCTCTATTCAT
TCTTTGTCGTTGCGCCTCCGAAACATGACCATTCCATTGTGTGTTCTGATATTCGAACTAGAAG
TAGAATTTTTTGGCACAAATTTTTTGGCCTATATCTTTGCCATTGATGCTTAACTTAACCTTGAT
TGCACCTCTAAGCACGATGTCAATGGTGGATACCCTTTGAAATACAATTCGAGCGAAAAGAG
ACTGATGGTTTGATGGGTGCTATACCTCCTGCATGTACAATACTTCAATGCAATCTCCATACGC
TATTGTCAAAAACTCTTTCTCTTTCACTTCCCTCCCCTCTCGTCCATTCTGTATCCTTTCGAAAA
CGTCACAAACATCGTCCTTGTCTCGTCTCTGCGAAAACACTTCACCACCACCACCACCGTAGTT
CCTCTTATCCATTTTAAAAAATTATTTAATTATAAAATTGTCCTATTTTAATTAAAAAATTAAT
TTTTATTAGATTAAATTAACTTAATTTAAAAAGGAACATTCCATTAAAATGTATCATATCATAA
AAAATAATTTATATGTTATTTAAAAAGAGTTGAATAGAATTTATCTAAATTTTAATATTTCTTA
ATTCAACTAATTTTAATATCATCTTTTACACTTTGAAAAACAATATTTTTTAAACAAAATAATA
AAAATAATCAAATTATTACAAAATTATTTTTTATTTGTATCTTTAGATTTTATATTTTTGAATTC
TAGTTCTGGTGAAATTTTTTCATATAAATAATTTTATCAAATTATTTCTAATTATATATTACAA
GTTTTAATTTTCTGAATCTTATTATTTTTTAATTTTTTTATTTCTTATGGTATTTTTTAACTCGAC
AAGATAAAAGATTAGTCAGCGGTATATTTAAACTATATTCAAGAATTTGTCTAACTAATAAAT
TGTTATATACATAAAACAAAAATTTAAACTTCCGATACTTACTTAAACAAATAAATAAATTAA
TAACTACTCTAATAACCAAATAAGTTAATCGACTAATCCAAATTTGTTTATTCCCTCTGTATTT
AGTAAAGAACAATGGCACCATATATAATGGTGTATTTCGCACGTGGGAGCTAATTAAGTTACT
CGAGCAATAATATATAGAGGTAGAGACTACCGC**ATG**CAAGTGCAAATTGA

Note: in promoter sequence, underlined regions are forward and reverse primers.
