# Supplementary material for: Genome-Wide Investigation of Apyrase (APY) Genes in Peanut (Arachis hypogaea L.) and Functional Characterization of a Pod-Abundant Expression Promoter AhAPY2-1p
Source: Int J Mol Sci. 2023 Feb 27;24(5):4622. doi: 10.3390/ijms24054622 (PMC10003104; doi:10.3390/ijms24054622)
Supplement: Supplementary file 1 [file ijms-24-04622-s001.zip › Supplementary Figures.pdf]

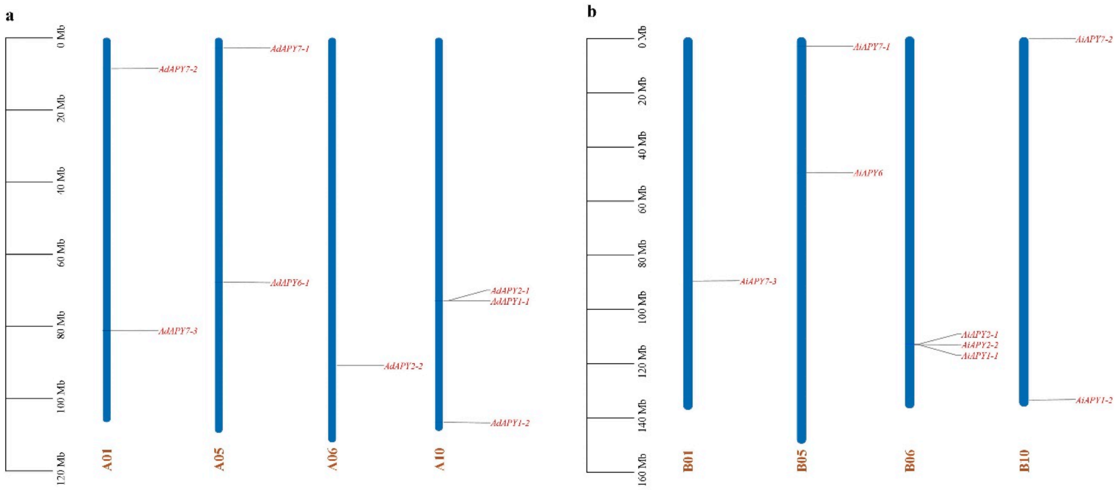

Figure S1. Chromosomal distribution of diploid peanut species. (a) Chromosomal distribution of *Arachis duranensis APYs*. (b) Chromosomal distribution of *Arachis ipaensis APYs*.

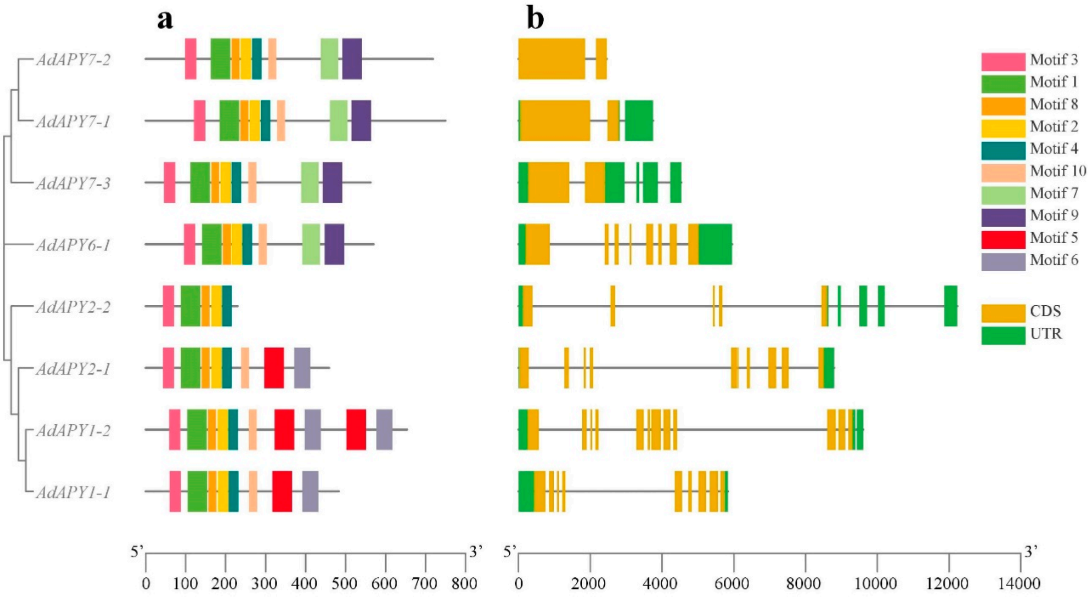

Figure S2. Conserved motifs and gene structure analysis of *AdAPYs*. (a) Top 10 conserved motifs of *AdAPYs* predicted by MEME suite. (b) Exon-intron distribution patterns of *AdAPYs*. The legends are given on right side.

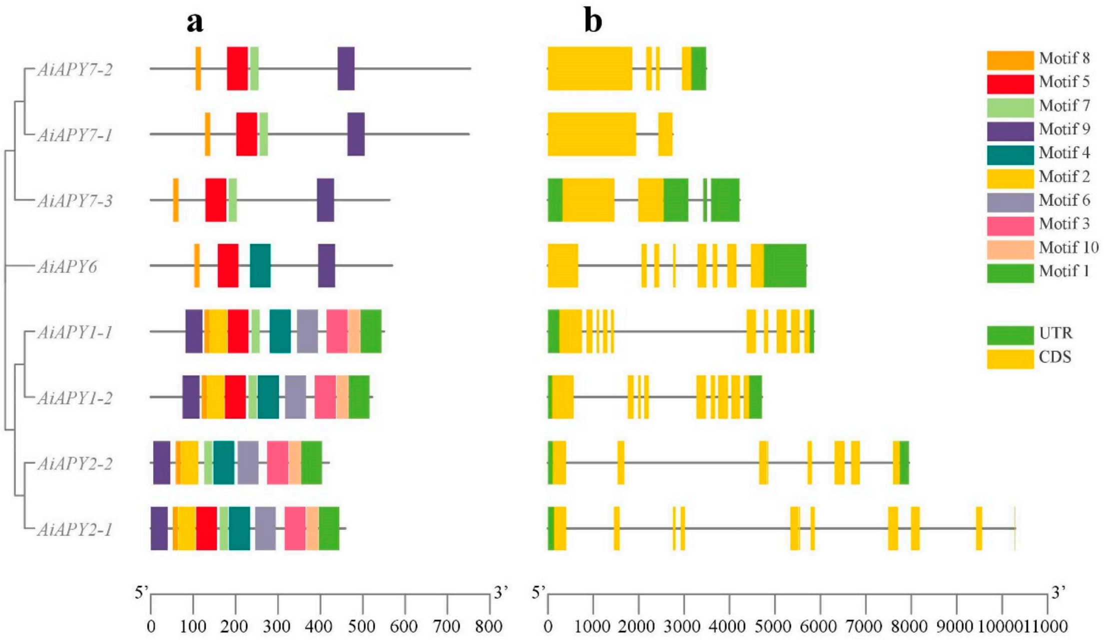

Figure S3. Conserved motifs and gene structure analysis of *AiAPYs*. (a) Top 10 conserved motifs of *AiAPYs* predicted by MEME suite. (b) Exon-intron distribution patterns of *AiAPYs*. The legends are given on the right side.

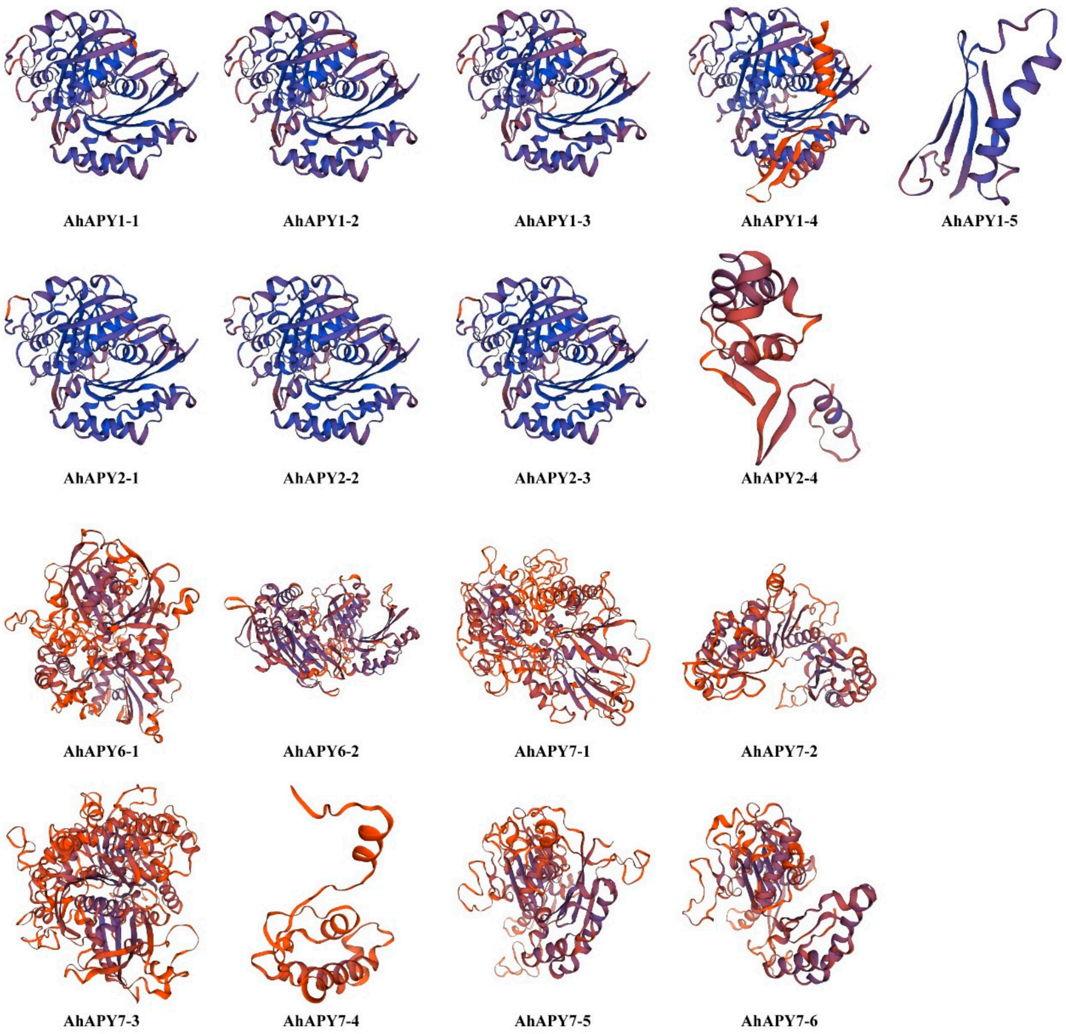

Figure S4. Simulation of 3D models of protein tertiary structures. The 3D models are simulated by Swiss-Model using the default parameters. As the proteins of the first and second group perform similar functions, 3D structures also showed similar simulations and proteins of group six and seven perform similar functions, so their 3D structural simulations are almost similar.

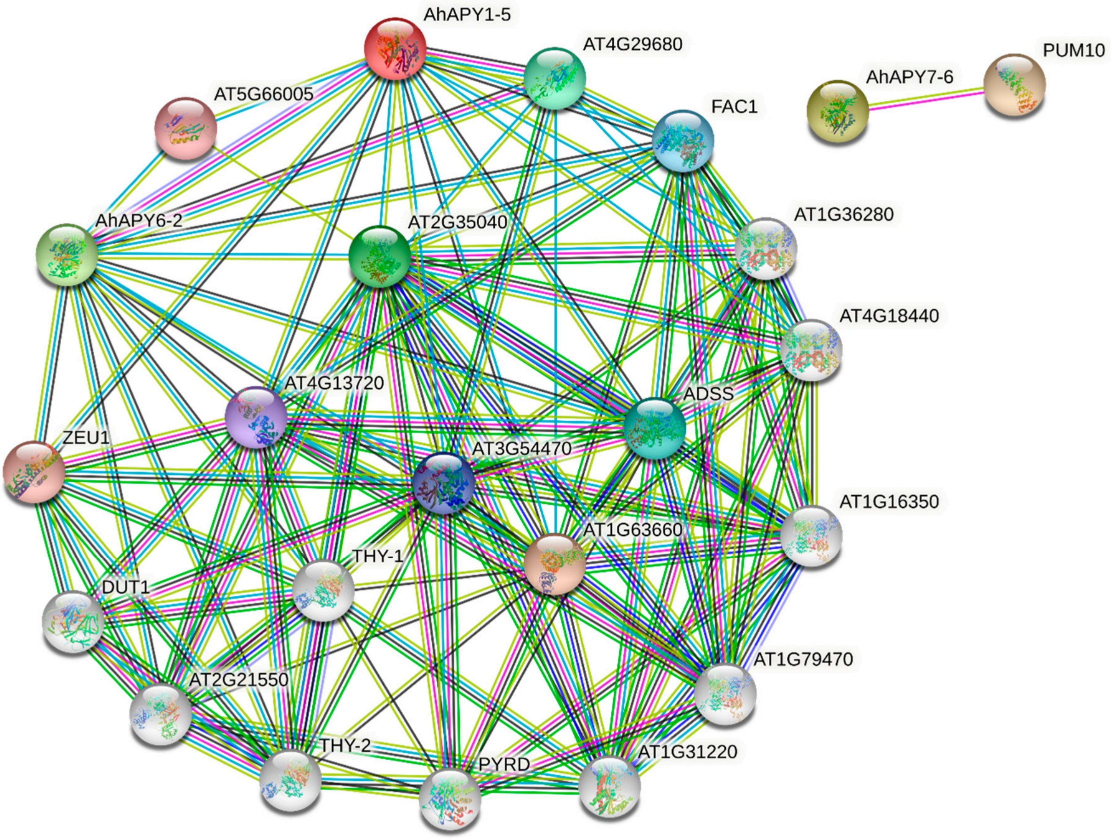

Figure S5. Prediction of protein-protein interaction network of *AhAPYs*. The interaction network showed that *AhAPYs* showed closely related functions with their homologs of Arabidopsis.

AhAPY2-1P

```
-2044 GATGAACTTT GTAATGGTCT GCGAGACAAC TTTTACATGA TGCAAAAGTG GAGCTAGAGG CGCGTCTAAA 1
                                      ABRE                  unnamed_4(-)   TATC-box
-1974 GAGGTAGGGA ACATGTGATA TATCCTGAAA ATGACGTGAG AATTATTTTA AGCGGACGAT ATATATCCCA 2
-1904 AACTAGATAC AATTATTTTC AATAAATTAA TCTTTTGATA TACAATATTC GTGAAAATGA ATTGTGTTTT 3
-1834 TATTCATCAT ACATAAGATC TGTTTGTTAA ATAAAATAAA ATTGTTTTTT AATAAACATA AGTCATACAA 4
                                 ERE          BOX 4
-1764 ATATTATTTG ATAAAATTAT TCTTAAAATT TAAAATTGAT TAATAGTGAA ATTCGTTCTT TAAAAATAAT 5
                                                             GATA-motif
-1694 TTATTTTTTA AATTAGTTTT TAAAAAATTA AATTAGTCAT ATTAGTTCTT AAAAGATAAG ATATAAGTCA 6
                            CGTCA-motif   ARE       G-Box          ABRE
-1624 AATTGGCCTT TCTATTAGTC AGATAATGAC GTGTCATATG AAATGCCACG TGTCATGATG ACGTGGTAGA 7
         BOX 4 unnamed_1                      G-Box(-)          o2-site(-)
-1554 TTAATACCAC GTAACATAAG ATGATTAGCC GATGTGTATC ACGTATTATC TAATATGTCA TAAATTTATT 8
            BOX 4                       Box III                     Box 4
-1484 TAAGTCAAAT TAATTCTAAA AGTACACACG CAAATCATTT TCATTATTAA AATTTTAAAA ATTAATCAAA 9
                  unnamed_6
-1414 TTAGTTCTTA TATAAATCTC TTTTATTTTT TTTATAATAT TAAATTTTAG ATAAATTCTA CTATACTCTT 10
                        WUN-motif
-1344 TTTAAAGTGA CATGTAATTG TAAATTACTC TGAGTGATCA TTTTATGATC GAATACTATC TTAATCATAA 11
-1274 TTAGGCCATT TTATAATTAA AAAATTATTT GAAAAGGGTG AATGTATAAT TTAATAAAAT ATTAAAAATT 12
                                                                        MYB
-1204 GAATCCTATT TTTTTTTTCA AATCAATCTT TTAAAAAATG TATCCTAACT CTCTTTAATG GTTGTCGCTC 13
  unnamed_4       TGA-element        LTR
-1134 CTTCTCTATT CATTCTTTGT CGTTGCGCCT CCGAAACATG ACCATTCCAT TGTGTGTTCT GATATTCGAA 14
                                   circadian (-)         unnamed_2 GT1-motif
-1064 CTAGAAGTAG AATTTTTTGG CACAAATTTT TTGGCCTATA TCTTTGCCAT TGATGCTTAA CTTAACCTTG 15
-994  ATTGCACCTC TAAGCACGAT GTCAATGGTG GATACCCTTT GAAATACAAT TCGAGCGAAA AGAGACTGAT 16
                         unnamed_4
-924  GGTTTGATGG GTGCTATACC TCCTGCATGT ACAATACTTC AATGCAATCT CCATACGCTA TTGTCAAAAA 17
  ARE (-)            unnamed_4                               CGTCA-motif
-854  CTCTTTCTCT TTCACTTCCC TCCCCTCTCG TCCATTCTGT ATCCTTTCGA AAACGTCACA AACATCGTCC 18
                                                                 ERE
-784  TTGTCTCGTC TCTGCGAAAA CACTTCACCA CCACCACCAC CGTAGTTCCT CTTATCCATT TTAAAAAATT 19
                                          AT1-motif
-714  ATTTAATTAT AAAATTGTCC TATTTTAATT AAAAAATTAA TTTTTATTAG ATTAAATTAA CTTAATTTAA 20
                                    AT1-motif
-644  AAAGGAACAT TCCATTAAAA TGTATCATAT CATAAAAAAT AATTTATATG TTATTTAAAA AGAGTTGAAT 21
-574  AGAATTTATC TAAATTTTAA TATTTCTTAA TTCAACTAAT TTTAATATCA TCTTTTACAC TTTGAAAAAC 22
                                                  AT1-motif
-504  AATATTTTTT AAACAAAATA ATAAAAATAA TCAAATTATT ACAAAATTAT TTTTTATTTG TATCTTTAGA 23
-434  TTTTATATTT TTGAATTCTA GTTCTGGTGA AATTTTTTCA TATAAATAAT TTTATCAAAT TATTTCTAAT 24
-364  TATATATTAC AAGTTTTAAT TTTCTGAATC TTATTATTTT TTAATTTTTT TATTTCTTAT GGTATTTTTT 25
-294  AACTCGACAA GATAAAAGAT TAGTCAGCGG TATATTTAAA CTATATTCAA GAATTTGTCT AACTAATAAA 26
                                           chs-CMA1a                 BOX 4
-224  TTGTTATATA CATAAAACAA AAATTTAAAC TTCCGATACT TACTTAAACA AATAAATAAA TTAATAACTA 27
         MYB
-154  CTCTAATAAC CAAATAAGTT AATCGACTAA TCCAAATTTG TTTATTCCCT CTGTATTTAG TAAAGAACAA 28
           TATA-box                ABRE                               CAAT-box
-84   TGGCACCATA TATAATGGTG TATTTCGCAC GTGGGAGCTA ATTAAGTTAC TCGAGCAATA ATATATAGAG 29
                     +1
-14   GTAGAGACTA CCGCATG 30
```

Figure S6. *Cis*-regulatory elements in *AhAPY2-1P*, predicted by the PlantCARE database.
